# Bench Testing of Sensors Utilized for In-Line Monitoring of Lubricants and Hydraulic Fluids Properties

**DOI:** 10.3390/s21248201

**Published:** 2021-12-08

**Authors:** Daniele Pochi, Renato Grilli, Laura Fornaciari, Monica Betto, Stefano Benigni, Roberto Fanigliulo

**Affiliations:** Consiglio per la ricerca in agricoltura e l’analisi dell’economia agraria (CREA), Centro di ricerca Ingegneria e Trasformazioni agroalimentari (Research Centre for Engineering and Agro-Food Processing), Via Della Pascolare 16, 00015 Monterotondo, Rome, Italy; renato.grilli@crea.gov.it (R.G.); laura.fornaciari@crea.gov.it (L.F.); monica.betto@crea.gov.it (M.B.); stefano.benigni@crea.gov.it (S.B.); roberto.fanigliulo@crea.gov.it (R.F.)

**Keywords:** condition monitoring techniques, sensors, viscosity, permittivity, relative humidity, electric conductivity, particle contamination, ferro-magnetic particles, agricultural tractors

## Abstract

This work reports the results of a study on the behaviour of five sensors recently developed for oil conditions monitoring, installed in-line in an experimental test rig for lubricants. The tests were carried out on seven oils of different origins (one synthetic ester, two bio-based synthetic esters, four vegetable oils) and use (two UTTOs and five hydraulic oils), under controlled working conditions, according to a specially designed test method. At first, the study concerned the identification of the conditions for the correct sensors’ installation. Then, the tests started applying to the fluids severe work cycles intended to accelerate oil ageing. The data of viscosity, permittivity, relative humidity, electric conductivity, particle contamination, and ferro-magnetic particles provided by the sensors were compared to the results of laboratory analyses made on oil samples taken during the tests with the aim of verifying the sensors measurements accuracy and reliability and selecting the more suitable ones to in-line oil conditions monitoring, in the perspective of introducing them also in field applications, e.g., on agricultural tractors, for preventing damages due to oil deterioration, and in managing the machine maintenance.

## 1. Introduction

In recent years, great efforts have been put into the development of diagnostic systems aimed at protecting plants and machines of various production sectors from damage. The diffusion of systems for predictive maintenance purpose was favoured by the availability of ever more performing sensors, from the point of view of miniaturization, accuracy, and reliability. Such characteristics make some of them suitable for in-line installation for real-time monitoring of the key components of plants and processes based, among the others, on transmissions and hydrodynamic machines. The integrity of the latter can be mainly safeguarded by monitoring the properties of the lubricants and hydraulic fluids used to operate them. Currently, such applications are extending also to the agricultural sector. Here, tractors, harvesters and, in general, all self-propelled machines carry out a wide range of operations based on the use of their transmission and hydraulic systems. Since most operations involve the natural environment, such as open fields, forestry, and orchards, the oil can be strongly contaminated by the dust, e.g., through the fittings during the connection between implements and tractors; at the same time, any oil leak due to failure would represent a source of pollution and of contamination of food and feed products. Consequently, to monitor the evolution of fluids characteristics and properties would help both to preserve the integrity and efficiency of hydraulic systems and to limit the impact of fluid dispersion in the environment.

Moreover, we should also consider the recently started research activity aimed at replacing petroleum-based lubricants with bio-based fluids [1,2,3]. The introduction of new lubricants in any production system requires experimental activity aimed at carefully assessing their suitability, preserving the machines and plants from damage, and the environment from dispersion of fluids (albeit bio-based). At first, the suitability of bio-based lubricants is usually assessed in proper work cycles based on special test benches, by monitoring the fluid properties through periodic samplings and laboratory analyses, capable of providing accurate information on the oil status [4,5]. The next evaluation step is based on the use of real machines (e.g., tractors) employed in normal work (e.g., farming operations), always monitoring the fluid status by sample analysis [6]. Such a method often requires the intervention of specialized (often external) laboratories and its limits consist of the costs, the time for analyses, and the time interval between samplings during which the fluid–machine system is exposed to undetectable variations or damage. Therefore, in both phases, the continuous monitoring of fluid conditions would be desirable. In this picture, several manufacturers are considering techniques for real time, continuous monitoring of fluids quality, for predictive maintenance purposes, by using suitable measurement sensors inserted along the flow lines [7,8,9]. These techniques help to provide early warning of machine failure and to minimize downtime, reducing maintenance costs, and to avoid bad lubrication conditions in the transmission and hydraulic circuits [10]. Such a non-destructive method would also contribute to prevent waste and environmental pollution, and to extend the lubricants life-time and increase the machines working availability. The above considerations can be extended to the sector agricultural machinery. Here, two main lubricant typologies are used: (i) Universal Tractor Transmission Oils (UTTO) and (ii) hydraulic fluids. The first are used in tractors as lubricants of transmissions, hydraulic power supplier and as heatsink [11]; the second works as a hydraulic power supplier in medium-high engine-power tractors with the hydraulic oil reservoir separated from the transmission box, in tractors with hydraulic transmission and, in general, in all machines equipped with specialized hydraulic systems [12].

In general, the reasons for lubricant deterioration are represented by oxidation, wear (metallic) particle contamination, non-metallic particle contamination, and water contamination. The various contaminants can work as catalysts of oxidative reactions, accelerating oil degradation [13,14]. The occurrence of said phenomena can be detected by monitoring a set of parameters describing the evolution of the main fluid characteristics, such as the Total Acid Number (***TAN***), the Kinematic Viscosity and the viscosity index, the water content, the amount of wear metals, and of contaminant particles. In the case of bio-based fluids, the Peroxide Value (***PV***) assessment can be introduced to get information on the presence of any primary oxidation process [15]. As said above, they can be accurately measured in a laboratory adopting specific standard methods.

As an alternative, several types of sensors can be used to evaluate oil conditions by monitoring and processing the data of various physical fluid characteristics (optical, electric, or magnetic) and correlating them to the chemical–physical parameters characteristic of the lubricants and hydraulic fluids. For instance, the relative permittivity describes the polar status of the fluid, which can be affected by the presence of water, contaminants, and product of oxidation; the electric conductivity is an oil’s specific parameter indicating contamination with other oils or substances; metallic particle contamination probably indicates machine wear and may result in overheating and component failure; and the presence of non-metallic particles can be caused by the bad sealing of some components against dust and debris from outside environment. The monitoring involves the entire oil volume circulating in the hydraulic system and can regard the above-mentioned parameters: Kinematic Viscosity [16,17]; water content [18]; wear metals [19]; particle contamination [20,21]; total acid number (***TAN***) [22]; and electric conductivity and relative permittivity [23,24]. Properly installed in machines and plants, along the flow lines of lubricants and hydraulic fluids, they operate continuous, real-time measurements of their chemical–physical properties, helping to evaluate their status [25]. Albeit less precise than laboratory analyses, the sensors seem effective to show early any variation in the key characteristic of the fluid allowing the selection of the most appropriate oil maintenance (refill) and replacement programs [26] and, ultimately, to prevent damage to machines, particularly in the case of the testing of new fluids like the bio-based ones. The sensors could also be part of an integrated system for monitoring the conditions of oil–plant systems, in which other techniques are adopted, such as, e.g., thermography, vibration analysis [27], or used to develop models for the prediction of the remaining oil life [8].

This work reports the results of a study on five recently developed sensors for oil condition monitoring installed in-line in an experimental test rig for lubricants [28], introducing a possible test method for the evaluation of the accuracy and reliability of these types of sensors. The tests were carried out on seven oils of different origins (one synthetic ester, two bio-based synthetic esters, and four vegetable oils) and technical characteristics (two UTTOs and five hydraulic oils), under controlled working conditions. At first, the study concerned the identification of the conditions for the correct sensors’ installation. Then, the tests on the fluids started applying severe work cycles intended to accelerate the oils’ ageing. For some parameters, the data provided by the sensors were compared to the results of lab analyses made on periodically taken oil samples. In general, such an approach should help to evaluate the accuracy and reliability of the sensors’ measurements and to select the most suitable to fixed plants and also to in-field applications, e.g., on tractors, by means of specific on-board monitoring systems that may support the operator in preventing damage caused by oil deterioration and in managing the machine maintenance.

## 2. Materials and Methods

The aim of the study was the comparison between the data provided by a series of sensors and the results of laboratory sample analyses in the measurement of certain characteristic parameters of lubricants and hydraulic fluids undergoing work cycles in a test rig. Such a system allows the application of controlled and repeatable working conditions to the tested fluids, detecting any variation in their technical performance (flowrate, pressure, power) relating to their status. The latter is commonly described by the trend of characteristic parameters resulting from the analysis carried out on fluid samples withdrawn periodically. In the present paper, such analysis data have been used to validate the online continuous measurements provided by a series of sensors installed on the test rig.

### 2.1. Parameters Considered in the Study and Relative Analytical Standards

- Viscosity—is probably the most important parameter for lubricants or hydraulic fluids and is commonly measured in laboratory as Kinematic Viscosity (cSt or mm^2^·s^−1^), according to the ASTM D445 standard method [29] by means of viscometers, calibrated, and thermo-stated tubes measuring the time required for the complete flow of a fixed oil amount. The viscosity values at 40 and 100 °C allow the calculation of the Viscosity Index, by means of tables reported in ASTM D2270 [30].
- Water content—The oil absolute water content is determined in laboratory, according to ISO 8534 standard [31], by means of Karl Fischer technique, using an automatic titrimeter (Metrohm, Herisau, Switzerland). The water content can also be measured as Relative Humidity (φ, %), which is calculated by means of the relation (1):
  (1) $$\varphi \;=\;100\;\cdot \;\frac{\rho_{w}}{\rho_{w,\max}}$$
  where *ρ_w_* is the water contained in the oil and *ρ_w_*,_*max*_ is the maximum amount of water dissolvable at the saturation limit. Since *ρ_w_*,_*max*_ is strongly temperature dependent, the relative humidity varies with the temperature.
- Electric conductivity—is a temperature dependent, oil specific parameter. Different fresh oils at the same temperature can be distinguished for their characteristic and common low values. The electric conductivity of the oil in a plant can vary due to oil changes and refills, to mixtures with other oils, and to contamination (with solids and liquids). An increase in oil conductivity is commonly associated with certain aging processes.
- Relative permittivity (relative dielectric constant)—is an indicator of fluid’s polarity. Base oils and additive packages from different manufacturers may differ in their polarity. The polarity and the course of the polarity of the fluid above the temperature are thus characteristics. The polarity of an oil can increases due to increasing content of water and contaminants [32] and/or to the aging process that results in polar products increase. If there is a change in the relative permittivity exceeding 10 to 20% compared to the fresh oil value, the oil should be carefully examined, especially if the rate of change in the signal increases significantly [33]. As the standard method IEC 60247:2004 [34] used in lab determination of permittivity and electric conductivity is specific for insulating fluids and seems unsuitable for the hydraulic fluids and transmission lubricants, a fluid check carried out on the viscosity, the acid number (TAN), and the peroxide number (for biobased fluids) could reveal significant increases in these parameters, confirming that an aging process is going on.
- Particle contamination—The level of contamination is determined by counting, within certain dimensional classes, the number of particles per fluid volume unit. The measurement is carried out by automatic particle counters that can be suitable to examine the fluid by sample or in-line. The contamination is then expressed as “Class of contamination”, according to several international standards described by their relative codes. The most adopted standards are the ISO 4406:2021 [35] and the NAS 1638 [36]: in this study we considered the former, whose classification is based on three dimensional classes: <4 μm, <6 μm, <14 μm. The level of oil contamination is described by codes (numbers 1 to 22) assigned to the oil depending on the number “***n***” of particles per mL of oil detected in each class: in the code “1”, the range of particle number per mL is 0.01 < n ≤ 0.02; in the code “2” the range is 0.02 < n ≤ 0.04. The range doubles at each step, until the range 20.000 < n ≤ 40.000 in the code “22”. Therefore, the oil status is described by three numbers. Despite the ISO 4406 codes ranging from “1” to “22”, the range most frequently considered in practical applications is that between “7” and “22”.
- Acidity and Total Acid Number (TAN)—Oil and fluid free fatty acids were evaluated by means of volumetric titration by phenolphthalein as an indicator, following the ISO 660 method [37]. A limit of 2 mg KOH g^−1^ has been established for TAN variation in hydraulic oils, according to the ISO 4263-3:2015 [38].
- Peroxide value—Oil peroxides value was determined by means of volumetric titration, based on the liberation of iodine from potassium iodide in the presence of hydroperoxides, according to ISO 3960 standard [39].
- Metal content analysis—According to ISO 21033 standard [40], extracted oil from oilseed crops and fluids sampled from the test bench were analysed for trace metals and phosphorous content by means of inductively coupled plasma atomic/optic emission spectrometry (ICP-OES) equipment (Perkin Elmer, Waltham, MA, USA) after dilution of samples in hydrogenated kerosene (Fluka, Buchs, Switzerland).

### 2.2. Sensors Used in the Tests

The sensors allow the measurement and the non-stop monitoring of a range of chemical–physical parameters, indicating the performance of hydraulic oils and lubricants. The sensors have ¾″ to 1″ threads, so that they can be installed on the hydraulic circuits either on the oil reservoir or directly on the return line, by means of a special line adapter that also has the function of normalizing oil flow. In any event, the operating pressure and temperature limits prescribed by the manufacturers must be respected. With reference to the parameters described in Section 2.1, all sensors selected for the test allow continuous, multiparametric measurements and can be used with different fluids without being re-calibrated.

**Figure 1:**
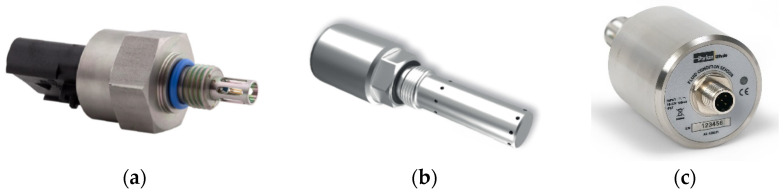
(**a**) Parker “FPS 2810” (***S1***); (**b**) Argo Hytos “LubCosH_2_O Plus II” (***S2***); (**c**) Parker Kittiwake “FCS” (***S3***).

- Sensor 1: “Parker FPS 2810” (***S1***)—The sensor is visible in Figure 1a and its main technical characteristics, and the measured parameters, are reported in Table 1. The sensor head features a patented tuning fork technology that detects and processes multiple physical properties of the fluid providing the measurements of its temperature, dynamic viscosity (cP or mPa·s^−1^), density, and relative permittivity. Dividing the dynamic viscosity by the density provides the Kinematic Viscosity (cSt or mm^2^·s^−1^), allowing the comparison with the data of laboratory viscometers.

**Table 1:** Main characteristics of the sensor Parker “FPS 2810” and measured parameters.

Measurement Ranges and Characteristics	Unit	Specification
Viscosity (dynamic)	mPa·s^−1^ (cP)	0.5–50
Accuracy for viscosity > 10 mPa·s^−1^	% Value	−5 to +5
Accuracy for viscosity < 10 mPa·s^−1^	mPa·s^−1^ (cP)	±0.2
Density	g cm^3^	0.65–1.65
Density Accuracy	% Value	−5 to +5
Permittivity	-	1–6
Permittivity Accuracy	% Value	−3
Fluid temperature	°C	−40 to 150
Temperature Accuracy	°C	0.1
Supply voltage (Peak)	Vdc	60
Ambient Operating Temperature (electronics)	°C	−40 to +125
Ambient Operating Temperature (fluid)	°C	−40 to +150
Storage Temperature	°C	−50 to +150
Input Current @12 VDC (In rush)	mA	<200
Operating Pressure	MPa	2.5
Vibration (Peak)	G RMS	20
- Sensor 2: Argo Hytos “LubCos H_2_O Plus II” (***S2***)—is shown in Figure 1b. It measures the fluid temperature (by means of a PT1000 platinum resistance sensor), the relative humidity (by means of a capacitive transducer), the electric conductivity, and the relative permittivity of the fluid, providing the corresponding values at reference temperature: relative humidity at 20 °C; relative permittivity at 40 °C; electric conductivity at 40 °C (Table 2).

**Table 2:** Main characteristics of the sensor Argo Hytos “LubCos H_2_O Plus II” and measured parameters.

Measurement Ranges and Characteristics	Unit	Specification
Measuring range		
Permittivity	-	1–7
Relative humidity	%	0–100
Electric conductivity	pS m^−1^	100–800.000
Fluid temperature	°C	−20 to +85
Measuring resolution		
Permittivity	-	1 × 10^−4^
Relative humidity	%	0.1
Electric conductivity	pS m^−1^	1
Fluid temperature	°C	0.1
Measuring accuracy		
Permittivity	-	±0.015
Relative humidity (10–90%)	%	±3
Relative humidity (<10 to >90%)	%	±5
El. conductivity (100 to 2000 pS/m)	pS m^−1^	±200
El. conductivity (2000 to 800,000 pS/m)	pS m^−1^	Typ. < ±10
Fluid temperature	°C	±2
Response time for RH measurement (0 to 100%)	Min	<10
Maximum operating pressure	MPa	5
Operating conditions		
Temperature	°C	−20 to +85
Relative humidity	% RH	0–100
Power supply	V	9–33
Max power input	A	0.2
Output		
Power output	mA	4 to 20
Accuracy power output	%	±2
Interfaces	-	RS 232/CAN open
Weight	G	140
- Sensor 3: Parker Kittiwake “FCS” (***S3***)—is shown in Figure 1c. The parameters measured by *S3* are relative permittivity, electric conductivity, relative humidity, temperature, and pressure. Table 3 reports the main characteristics of ***S3*** [41].

**Table 3:** Measured parameter and main technical characteristics of ***S3***.

Measurement Ranges and Characteristics	Unit	Specification
Measuring range		
AC conductivity	pS m^−1^	0 to 999
Permittivity	-	0 to 8
Relative humidity	%	0 to100
Working pressure	MPa	−20 to +100 °C
Temperature of fluid	°C	0 to 1
Measuring accuracy		
AC conductivity	%	±5
Permittivity	%	±5
Relative humidity	%	±2
Working pressure	MPa	±0.5
Fluid temperature	°C	±0.02
Maximum fluid pressure	MPa	1
Ambient operation temperature	°C	−20 to +80 °C
Ingress protection	-	IP67
Communication	-	Modbus over RS485
- Sensor 4: Parker “I count PDR” (***S4***)—By means of a laser diode optical detector, this sensor (Figure 2a) continuously measures particle contamination, averaging the values in a 5 to 180 s adjustable measurement period (pre-set value: 60 s) [42]. ***S4*** output can be chosen between variable current (4.0–20 mA) and variable voltage (0.3–4.8 VDC). In both cases, for each dimensional class, the signal determined by the detected particle number is converted into the corresponding standard ISO 4406 code (“***0***” to “***22***”). Alternatively, the NAS 1638 classification mode can be selected obtaining the related NAS codes. ***S4*** also provides the measurement of fluid relative humidity. Table 4 reports the main characteristics of the sensor. It requires accurate flowrate control, which was achieved by installing, downstream of ***S4***, the controller PARKER “IPD Flow Control Device”, shown in Figure 2b, according to manufacturer recommendations.

**Figure 2:**
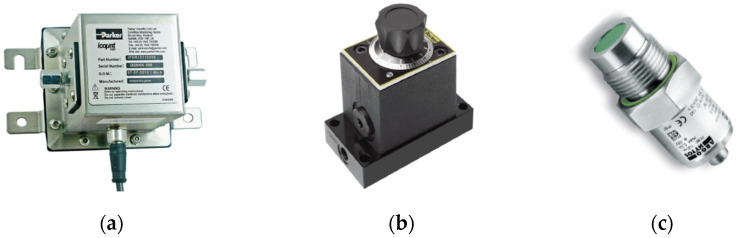
(**a**) Parker “I count PDR” (***S4***); (**b**) Flowrate controller for ***S4***; (**c**) Argo Hytos “Op Com FerroS” (***S5***).

**Table 4:** Measured parameter and main technical characteristics of ***S4***.

Measurement Ranges and Characteristics	Unit	Specification
Product start-up time (minimum)	s	5
Measurement period	s	5–180
Reporting interval (via RS232)	s	0–3600
Reference International codes	-	ISO 7–22, NAS 0–12
Particle limits	-	19/18/15
Working pressure	MPa	0.2–42
Flow range through icount PDR	mL min^−1^	40 to 140 (optimum 60)
Online flow range via System 20 sensors	l min^−1^	6–25
Ambient storage temperature	°C	−40 to +80 °C
Environment operating temperature	°C	−30 to +60 °C
Fluid operating temperature	°C	+5 to +80 °C
Operating humidity range	% RH	5–100
Moisture sensor stability (at 50% RH in 1 year)	% RH	±0.2
Power requirement	VDC	9–40
Current rating	mA	120
Certification	-	IP69K rating
- Sensor 5: Argo Hytos “OPCom FerroS” (***S5***)—The sensor (Figure 2c) measures the wear of mechanical components by detecting ferromagnetic particles. Through the early detection of wear, ***S5*** should contribute to optimize service measures planning, minimizing damages and downtimes [43]. The sensor detects the number of ferromagnetic particles that accumulate at the permanent magnet of the sensor head. ***S5*** can distinguish between fine particles (in the micrometre range) and coarse ferromagnetic fragments (“chunks”, in the millimetre area). The output signal ranges from 0 to 100% and indicates the occupancy of the sensor surface with ferromagnetic particles or fragments. When 100% occupancy is reached, an automatic cleaning function compensates the magnetic field of the magnet, causing the particles detachment from the sensor head. The main characteristics of ***S5*** are reported in Table 5.

**Table 5:** Measured parameter and main technical characteristics of ***S5***.

Measurement Ranges and Characteristics	Unit	Specification
Measuring range		
Fine particles	%	0–100
Coarse particles	-	1
Measuring resolution		
Fine particles	%	0.1
Coarse particles	-	1
Max. operating pressure	MPa	2
Operating conditions		
Temperature	°C	−40 to +85
Relative humidity	% RH	0–100
Min. distance for attraction of fine particles (1 g)		
in oil with Kinematic Viscosity <100 mm² s^−1^	mm	≅9.0
in oil with Kinematic Viscosity 300 mm² s^−1^	mm	≅7.5
in oil with Kinematic Viscosity 500 mm² s^−1^	mm	≅7.0
Min. flow velocity for automatic cleaning	m s^−1^	0.05
Max flow velocity	m s^−1^	1
Protection class	-	IP67
Power supply	V	9–33
Interfaces	-	RS 232/CANopen
Weight	g	140

**Figure 3:**
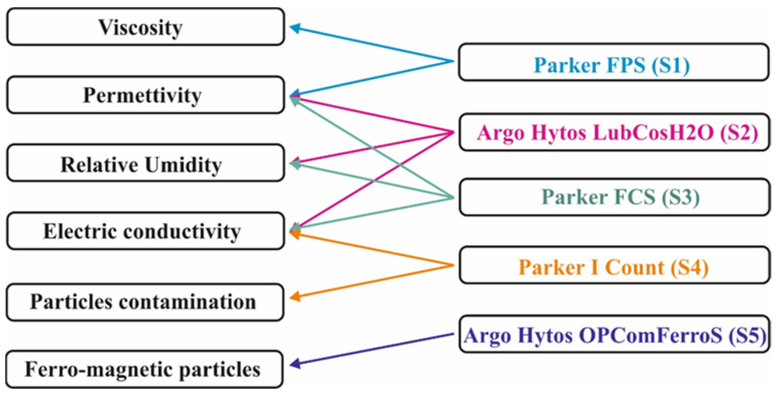
Parameters and sensors: some parameters are measured by more sensors.

Figure 3 provides an overview of the relationships, within the tests, between the five sensors and the parameters described in Section 2.1. It can be noticed that some parameters are measured by more sensors; thus, beyond the comparison between the data they provided and the laboratory results, it could be possible the comparison among sensors.

### 2.3. Test Conditions

**Figure 4:**
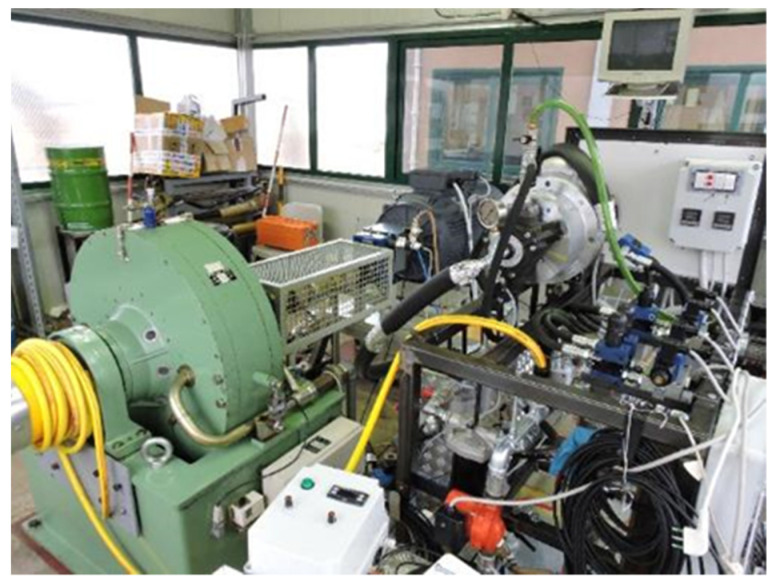
The test rig installed in the tractor test laboratory at CREA.

The sensors were installed and tested in a test rig (Figure 4) developed at CREA for comparative tests on hydraulic fluids, on lubricants for transmission and on UTTO fluids (Universal Tractor Transmission Oil).

The test rig was designed to apply heavy work cycles (through full control and repeatability of thermal and working parameters) to a small oil volume, with the aim of accelerating the aging process. Considering the total energy flowing through the oil volume unit (hydraulic and mechanic work, and thermal energy dissipated) a 150 h work cycle in the test rig corresponds to about one year (800–1000 h year^−1^) of average use of a medium power agricultural tractor [44]. In other words, the fluid-aging rate in the test rig is increased 5 to 6 times related to normal working conditions [45].

The test rig consists of a main circuit, including an oil reservoir, a low-pressure circulation pump, a main filter, and a heat exchanger. Two independent operating circuits are inserted onto the main circuit and are connected to the same circulation pump: (1) Hydraulic circuit—Its function is to apply hydraulic loads to the oil by means of a high-pressure radial piston pump and a distributor block equipped with solenoid valves, for sending the oil to overpressure valves (preloaded at 10, 20, 30, 40 MPa) but also in free flow. (2) Mechanical transmission circuit—Its function is to apply mechanical stress to the lubricant, such as those applied by the power transmission of agricultural tractors (gearbox group). In this case the stress is achieved utilizing an axial speed multiplier (transmission ratio: 1/2.44) with a pair of helical gear wheels placed in an oil bath. This multiplier receives the oil from the circulation pump at about 60 °C and sends it, at about 85 °C, toward the heat exchanger and then back to the oil reservoir. The “transmission circuit” fully functions when a resistant torque is applied to the secondary shaft. For this purpose, the rotary multiplier was placed between an asynchronous electric motor (45 kW of power at 980 min^−1^), which operates its primary shaft, and a torque dynamometer used to apply the resistant torque to the gear wheels. By controlling the rotation speed of the secondary shaft and the torque delivered, it is possible to apply the desired power, making the system perform specific and repeatable workloads. The two circuits just described can work singularly (for testing of transmission lubricants or hydraulic fluids), or together, for testing of multi-function oils such as the UTTOs. The different plant sections are equipped with thermocouples, pressure gauges, and connected alarms, for continuous monitoring of the most critical parameters (oil temperature and pressure), which must remain within the operating range of the most sensitive components.

Table 6 shows the working conditions applied by the test rig to the oils, both in the hydraulic and the transmission section. The reported data of work and energy have been calculated basing on the above-mentioned test interval of 150 h as a reference.

### 2.4. Installation of the Sensors in the Test Rig

**Figure 5:**
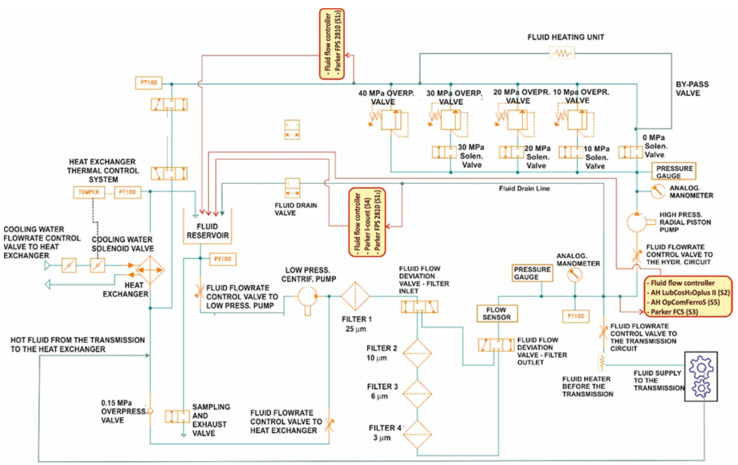
Positions of the sensors along the fluid lines of the test rig. The sensors are grouped in the yellow boxes, while the oil in- and outflow are described by red lines.

Figure 5 shows a sketch of the test rig and of the positions where the sensors have been installed. It can be noticed that two sensors ***S1*** were installed in the tests: ***S1_1_*** in the low-pressure section, and ***S1_2_*** at the high-pressure section outlet.

**Figure 6:**
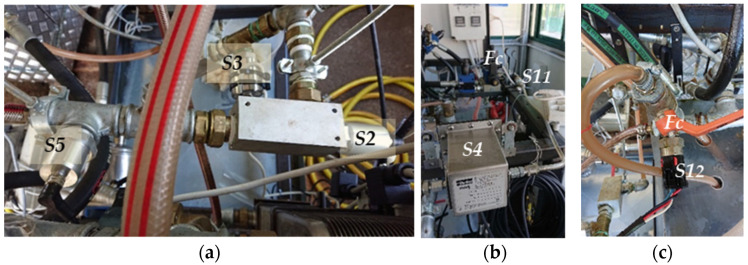
Sensors installed in the test rig: (**a**) secondary line supporting ***S2***, ***S3***, and ***S5*** and flow controller (not visible), derived from the main oil delivery line; (**b**) secondary line supporting ***S1_1_*** and ***S4*** and the flow controller, ***Fc*** derived from the main oil delivery line; (**c**) line supporting ***S1_2_*** and ***Fc***, derived from the 40 MPa valve outlet and terminating directly in the reservoir.

Two main groups of sensors have been installed in the low-pressure section. The first group (Figure 6a) consisted of ***S2***, ***S4***, and ***S5*** and a flowrate control valve, downstream of the sensors. The second group (Figure 6b) was formed by ***S1_1_*** and ***S3*** and a flow controller valve. Both groups receive the oil from the drain line. The oil exiting from both groups goes directly in the reservoir. The very low sensors oil inlet did not sensitively affect the flowrate in the delivery line required by the plant to correctly work. A third group (Figure 6c), only consisting of ***S1_2_*** and a flow controller valve, was installed in the 40 MPa valve outlet line (direct to the heat exchanger), for the evaluation of the sensor behaviour with high temperature oil. Additionally, in this case, the oil inlet to the sensor was obtained by draining a small oil flow, while the outlet oil was sent directly to the tank.

### 2.5. Oils Used in the Tests with the Sensors

The behaviour of the sensors was monitored during the test of seven oils:

- Two UTTO. One commercial oil, ***A***, largely used in the transmissions and hydraulic systems of agricultural tractors; one experimental synthetic ester, ***B***, based on vegetable oil.
- Five hydraulic fluids. One experimental synthetic ester, ***C***, based on vegetable oil; two supplies, ***D1*** and ***D2***, of the same refined high erucic vegetable oil, which differ in their respectively lower and higher concentrations of antioxidant (for food use); two supplies, ***E1*** and ***E2***, of the same refined high oleic vegetable oil, with additives as ***D1*** and ***D2***.

The main characteristics of the oils are reported in Table 7.

### 2.6. Test Methodology

The oil test method adopted was based on the application of the test conditions described in Table 6 for the entire duration of the test, divided into test days, considering a daily test time ranging from 7 to 10 h per day. The duration of the tests varied depending on the properties of each oil and its use, which influenced the onset of aging processes in different ways. During the work cycles, oil samples were withdrawn and analysed to detect any chemical–physical changes in the oil under test, with reference to the parameters described in Section 2.1. The results of laboratory analyses were compared to the average daily parameters values measured by the sensors on the same days of the withdrawal of oil samples, with the aim of evaluating the quality of measurement and attitude of the sensors to such types of applications.

### 2.7. Data Collection and Analysis

The reference data used to validate the measurements provided by the sensors came from the analyses of oil samples carried out at CREA laboratory and by three external specialized laboratories (“LMP srl”, Guidonia, Rome, Italy; “MECOIL srl”, Florence, Italy; INNOVHUB—Stazioni Sperimentali per l’Industria, Milan, Italy). The average time interval between two oil samplings was 30 h, but sometimes it could be different depending on test trend and duration. Table 8 shows which sensors and Lab-data were used in each oil test.

Regarding the acquisition of the signals sent by the sensors, a power and control panel receives all the signals from the different sensors and makes them homogeneous for acquisition and processing. This panel also manages the delivery mode (sequence) to the acquisition system, the alarms, and the eventual system shutdown in case of failures that could damage the sensors (e.g., oil flow interruption). The data acquisition and recording were made by means of a PC, in which dedicated software allowed continuous system status display during the test and automatic data storing at the end of a day of testing (file extension “.txt”). The data matrices were copied and processed in the pre-set spreadsheets (one for each trial day) of a Microsoft Excel file, where, for each parameter, the following daily summary values were calculated: test time, mean, maximum and minimum values, standard deviations, and standard error. In the case of temperature dependant parameters (viscosity, permittivity, electric conductivity, and relative humidity), the standard error only refers to values measured at constant temperature (corresponding to the operative temperature of the section in which each sensor was installed).

As to the temperature, the data provided by the sensors were compared to those of two “PT 100” thermocouples installed in the test rig. One was installed in the low-pressure oil delivery line (operative temperature upper limit: 60 °C). Here, very small differences were always observed for all sensors, but ***S1_1_***, as a consequence of the different positions of sensors and thermocouples, which were differently affected by the thermal exchange between the oil and external environments. As above described (Figure 6b), ***S1_1_*** was installed apart from the other sensors that measure the temperature, in such a way as to allow the temperature to be maintained at 40 °C. The second thermocouple was installed at the outlet of the 40 MPa overpressure valve, where it monitors the temperature that the oil reaches in the high-pressure section, which is constantly 99–100 °C. In this case, the sensor involved, ***S1_2_***, is installed about 40 cm downstream from the outlet, where the temperature is affected by oil cooling. Periodical controls by means of an immersion temperature probe confirmed the correctness of ***S1_1_*** and ***S1_2_*** temperature values. On the basis of these considerations and the stability of the temperatures observed in the different sections throughout the tests, the measurements provided by the sensors can be considered sufficiently correct in the framework of this study. Therefore, the discussion below will only concern the results relating to the parameters reported in Figure 3, which best describe the oil condition. Eventually, the duration of the seven work cycles varied oil by oil, depending on their characteristics and behaviours.

### 2.8. Evaluation of the Sensors

In the case of chemical–physical parameters which were the subject of lab analysis, the sensors daily summary values observed in the days of oil samplings were compared to the lab results in order to refer all data to the same oil conditions. Such a comparison was repeated for all samplings made on each oil, obtaining the series of values that describe the evolution of each oil condition all along the single test. For each parameter measured by several sensors (permittivity, and relative humidity), the entire sets of average daily values were used both with reference to laboratory tests where available, and for a comparison between sensors, in order to evaluate also their consistency and reliability through mutual validation.

The lab and sensors data were accompanied by the uncertainty of measurement. Due to the high number of data, it was very difficult to report all uncertainty values or to represent them in diagrams. So, they were synthesized in average percent values respecting the homogeneity of the data used in the calculations. As to the lab data, for each parameter, the uncertainty values declared in the certificates of analysis were calculated as a percentage of the measured values, and the average percent uncertainty was reported.

As regards the data provided by the sensors, the average extended uncertainty, ***Ū(x)_%_***, was reported. ***Ū(x)_%_*** was assessed, according to the standard ISO/IEC GUIDE 98-3:2008 [46], based on the sensors accuracy ***u(x)_%S_*** and the error in measurement ***u(x)_%M_***. The accuracy of ***S1***, ***S2***, and ***S3***, declared in their datasheets (with probability *p* = 95%), is reported in Table 1, Table 2 and Table 3. It is often expressed as “±% of value”. When the accuracy was declared in the same parameter’s unit measure, the corresponding “±% of values” were calculated for each oil and parameter, on the daily average values resulting from sets of values observed at constant oil temperature (operative temperature in the section of the test rig in which each sensor was installed). The uncertainty due to the error in measurements ***u(x)_m_,*** was obtained through calculation of the standard deviation “s” on the same daily sets of values at constant temperature. The interval “±2s” was considered to define the uncertainty in measurement with *p* = 95%. Moreover, in this case, it was expressed as “±% of value” [***u(x)_%M_***], where the value was the daily average. The accuracy values of ***S4*** and ***S5*** were not reported by the manufacturers.

Eventually, the average extended uncertainty, ***Ū(x)_%_***, was calculated for each oil by means of the relation (2):

***Ū(x)_%_= ū(x)_%S_ + ū(x)_%M_*** (2)

where

***ū(x)_%S_*** is the average uncertainty of sensors at 95% of probability, as above defined;

***ū(x)_%M_*** is the average uncertainty of measurement, resulting from the daily ***u(x)_%M_*** obtained in each oil test. It takes in account all causes of variability in measurements (sampling error; small temperature variations though not recorded by the sensors; and the effect of the instrumental chain and data processing).

For the visual evaluation of the aforementioned comparisons, the relative trends were represented in diagrams, while the correspondence between the series under comparison was statistically assessed, by series pairs (e.g., ***S1*** vs. ***lab***, ***S1*** vs. ***S2***, etc.), by means of the Pearson test, providing the correlation coefficient, ***r***, and the probability, *p*, of non-correlation. Such statistical elaboration was carried out by means of the software “Past 4.05”.

### 2.9. Additional Specific Test on Sensor 5 (S5)

**Figure 7:**
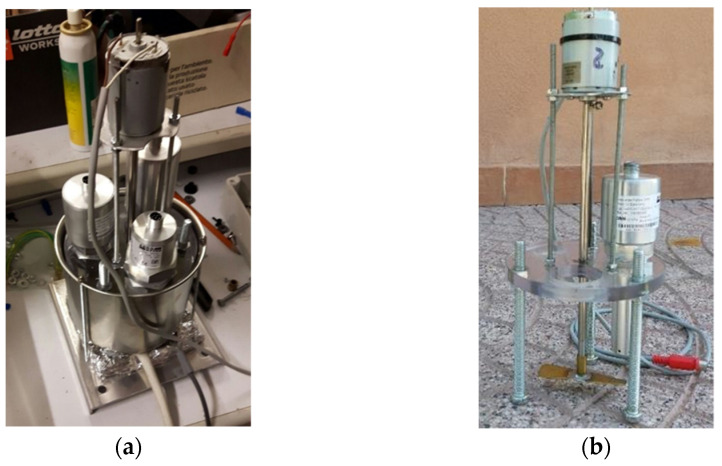
(**a**) The test bench for sensor with associated adjustment and control units. (**b**) Detail of the cover provided with the appropriate holes for housing the three sensors and the electric motor that drives the system for mixing the fluid.

The test aimed at better evaluating the effectiveness of ***S5*** (Argo Hytos “OPCom FerroS”) to detect the presence of known amounts of ferromagnetic particles added in different steps to a given oil volume. On purpose, a specific piece of equipment was used (Figure 7), which consists of a stainless-steel vessel for the oil (volume: 1 dm^3^) featuring a thermostatic resistance at its bottom, for oil heating. A specific cover, with proper holes, can lodge the sensors (up to three), allowing their sensing heads to remain immersed in the oil. The cover is equipped with a fluid stirring system, needed to avoid fluid stratification in different temperature zones, so that the installed sensor can work properly. In this test, the average temperature of the fluid was set n at 58°C, measured at the reservoir outlet, approximately the same value in the hydraulic circuit of the test bench used in the seven test above described.

Such an equipment was used to carry out a controlled iron (Fe) contamination test of a synthetic UTTO lubricant in which ***S5*** was immersed. The contamination was determined by adding to 1 dm^3^ of oil subsequent amounts of iron oxide powder (Fe_2_O_3_; maximum dimension: 44 mm; purity: 99%). Starting from new oil, seven contamination steps were carried out. After each step, ***S5*** was left working for 30 min and the data of the occupancy rate (fine fraction, chunks, and total) were recorded obtaining the trend of this parameter at increasing Fe_2_O_3_ concentration in the oil. Furthermore, in this case, the series of the occupancy rates average values obtained at each step (fine fraction and total) were compared to the series of Fe concentrations in the oil by means of the test of Pearson test to verify the presence of correlation among them.

## 3. Results and Discussion

### 3.1. Uncertainty in Measurements

To better evaluate the sensors and the test results, and compare them to the results of lab analyses, the data were accompanied by the uncertainty in measurement assessed as described in Section 2.8. Table 9 reports the uncertainty, with 95% probability, declared by the laboratories for the analyses they carried out.

**Figure 8:**
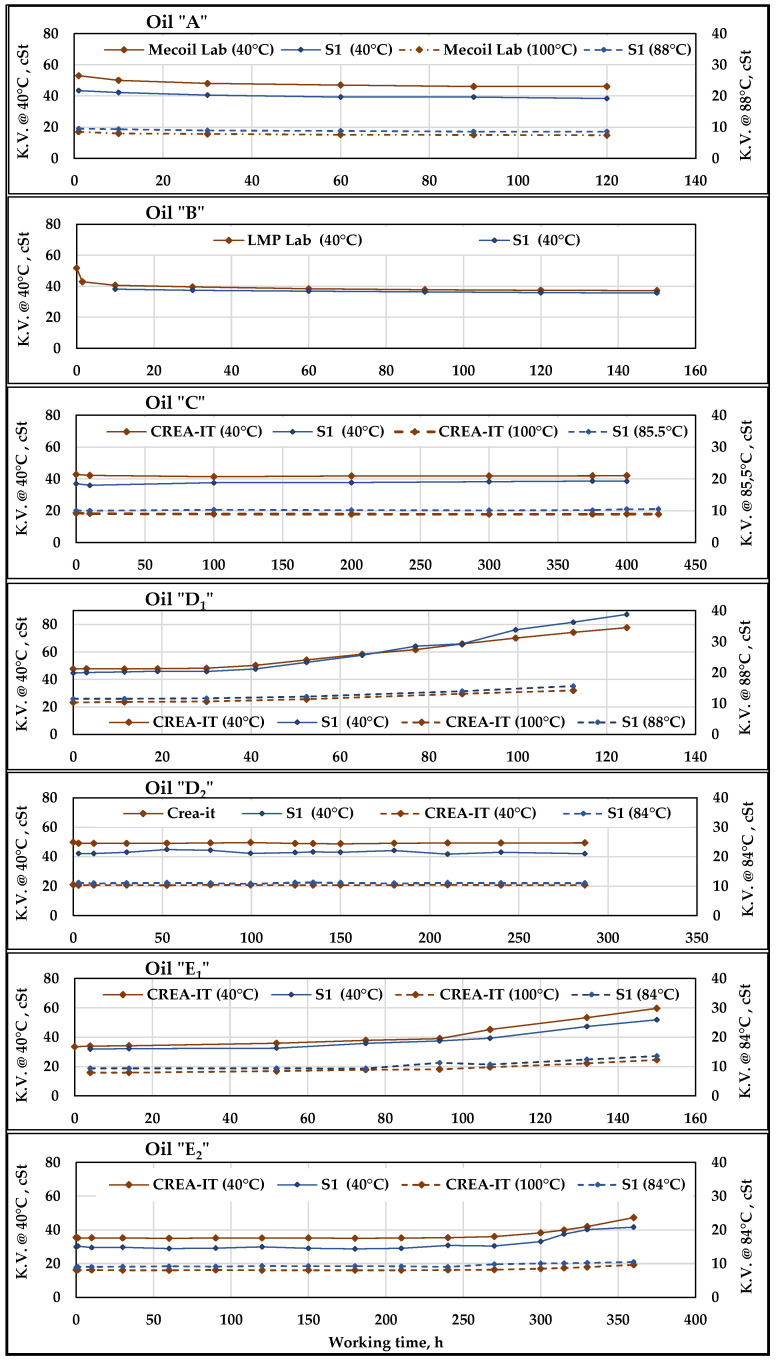
Kinematic Viscosity (K.V.) measurements provided by ***S1*** compared to the Lab-data at 40 and 100 °C. The diagrams refer to the seven oils subjected to working cycles of different duration. ***S1_1_*** measurements were made with oil at 40 °C while ***S1_2_*** provides the highest temperature values observed at the 40 MPa overpressure valve outlet (indicated in the legenda).

**Figure 9:**
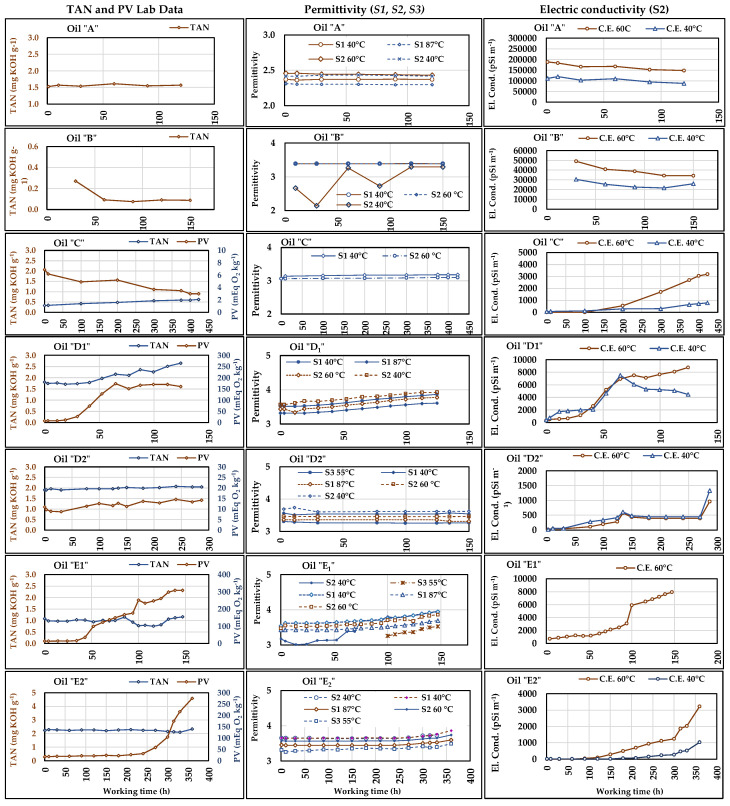
Trends of the permittivity measured by ***S1***, ***S2,*** and ***S4*** and of the electric conductivity (EC) measured by ***S2***, both compared to the Lab-data of ***TAN*** and ***PV***. The diagrams refer to the seven oils subjected to working cycles of different duration. The two ***S1*** worked with oil at 40 °C and at high temperature (about 87 °C) at the 40 MPa valve outlet; ***S2*** and ***S4*** data refer to the actual oil temperature in the delivery section (around 60 °C). ***S2*** also provided the permittivity and E.C. values measured at the actual oil temperature in the delivery section (values indicated in the legenda) and the calculated values at 40 °C.

**Figure 10:**
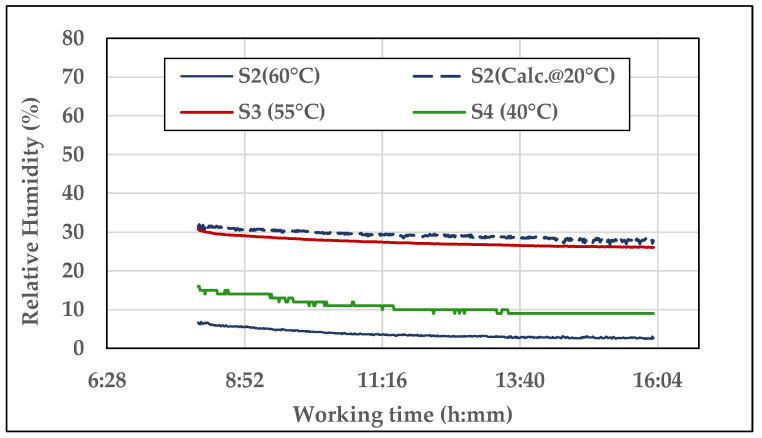
Example of the trends of the Relative Humidity measured by ***S2***, ***S3,*** and ***S4*** during one day of test (day 4, oil ***E2***, time of test: about 9 h).

**Figure 11:**
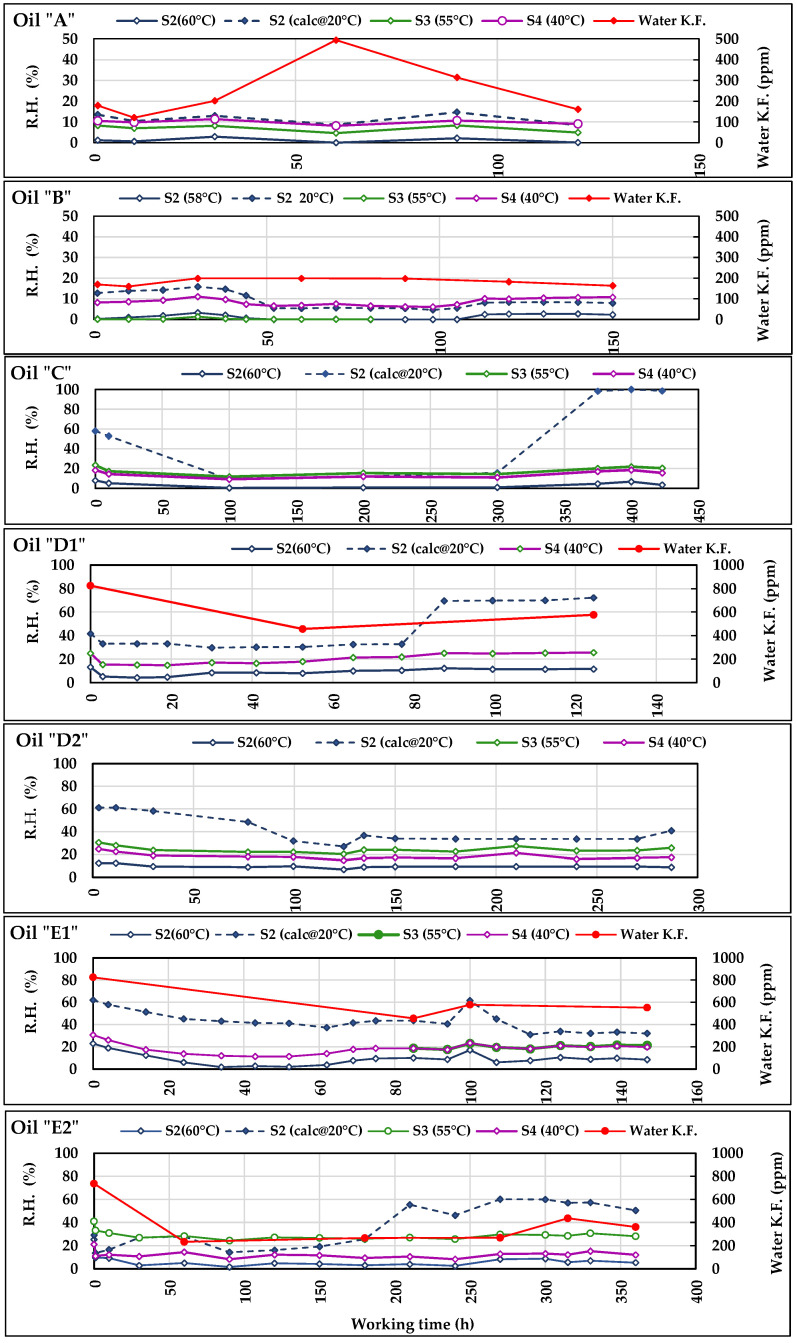
Trends of the Relative Humidity measured by ***S2***, ***S3,*** and ***S4*** during the tests with the seven oils. In the diagram of oils ***A***, ***B***, ***D1***, ***E1***, and ***E2***, RH trends are compared to those of the water content measured in laboratory according to Karl Fisher method.

**Figure 12:**
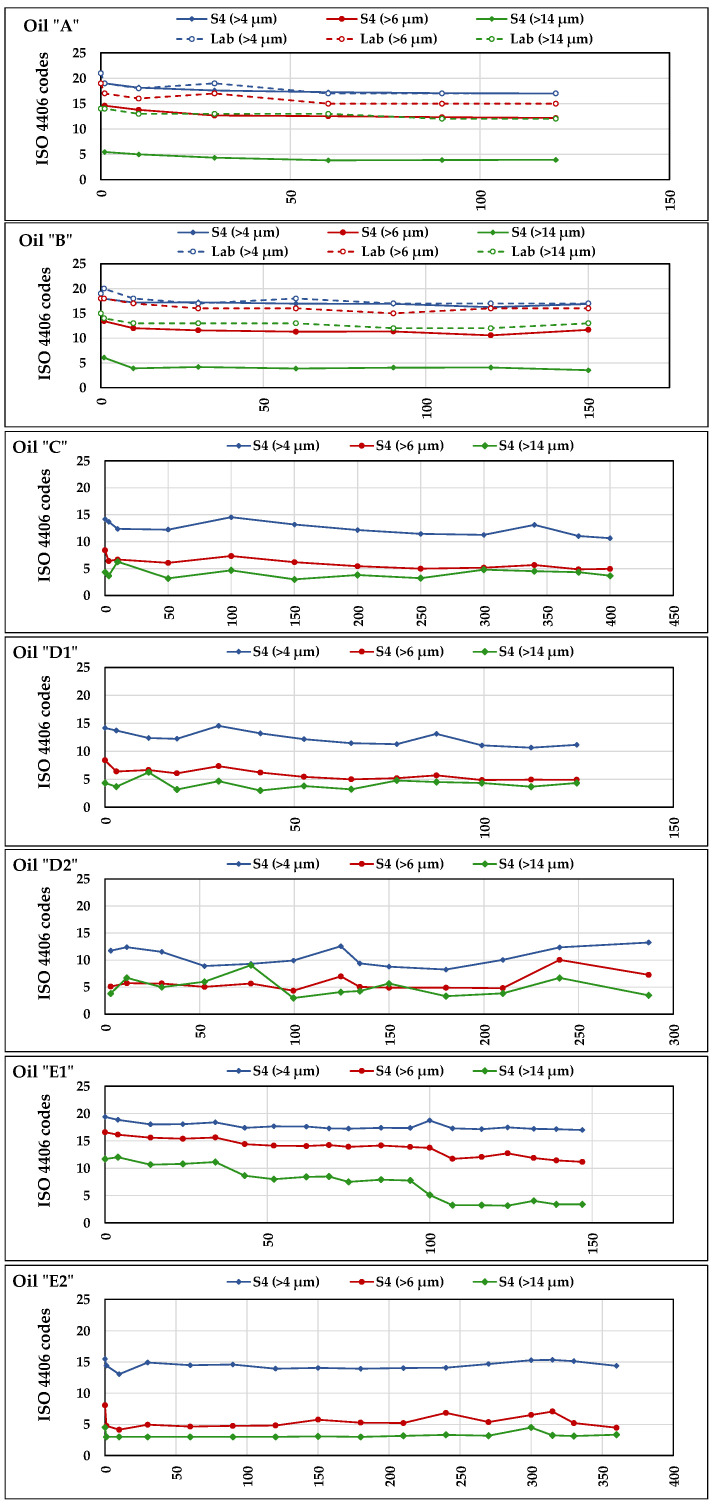
Trends of the ISO 4406:2021 particle contamination codes provided by lab analyses (for the oils ***A*** and ***B*** only) and by ***S4*** (for all seven oils).

In Table 10 are reported, for each oil test, the average extended uncertainty, ***Ū(x)_%_***, assessed for the Kinematic Viscosity(K.F.), permittivity, electric conductivity, and relative humidity (***RH***). As explained in Section 2.8, the assessment of ***Ū(x)_%_*** was carried out referring to constant oil temperature conditions to limit the effects of temperature variations on said parameters. Therefore, the temperatures considered were those measured by each parameter in the relative section under stable working conditions. They are indicated in the legends of Figure 8, Figure 9, Figure 10, Figure 11 and Figure 12. Each value forming the series represented in the diagrams of said figures should be considered. The accuracy of ***S4*** for particle contamination was not indicated in the datasheet. As said above, the sensor describes the level of contamination by means of standard codes, as the real measurement consisting of the number of particles is not provided. The accuracy of ***S5*** for ferromagnetic particles was also not reported, and the extended uncertainty could not be calculated for ***S4*** and ***S5***.

### 3.2. Viscosity

The results of the viscosity measurements, both by the sensor ***S1****,* and the laboratory involved time by time, are shown in Figure 8 for the seven oils described in Section 2.5. Each viscosity value should be accompanied by the relating uncertainty value reported in the Table 9 and Table 10, respectively for laboratories and sensor. Despite their higher extended uncertainty, which ranges from 6.18% to 9.54%, the sensor ***S1*** seemed to be capable of correctly monitoring the trend of the viscosity.

The lab viscosity was measured at 40 and 100 °C. ***S1_1_*** measurements at 40 °C were possible by suitably adjusting the flowrate in the related section (Figure 6b) in order to allow the oil to cool to the lower ambient temperature, passing from about 60 °C, typical for the delivery line of the test rig, to the new thermal equilibrium point of 40 °C, with very small oscillations. As to the oil at high temperature, despite the thermocouple, installed at the very outlet of the 40 MPa overpressure valve, normally indicate temperature levels of 99–100 °C, the viscosity measurements were made by ***S1_2_*** about 40 cm downstream and suffered the same oil cooling phenomenon just described for ***S1_1_***. Thus, the average temperature of the oil entering varies from 84 to 88 °C, depending on the ambient temperature. The ***S1_2_*** viscosity values and the Lab-data at 100 °C were, however, compared to evaluate the behaviour of the sensor under high temperature conditions.

The diagrams of Figure 8 show that in Oil ***B*** test, ***S1_2_*** measurements were not carried out, due to a sensor failure, while ***S1_1_*** measurements at 40 °C started at 10 h working time. In the Oil ***D2***, ***S1_1_*** data collection started after 90 h from test start, due to problems in the data acquisition system.

Despite some differences in the viscosity values, those provided by both ***S1_1_*** and ***S1_2_*** have trends similar to those of the Lab-data. It can be observed that the related curves are parallel, describing the evolution of the parameter during each test depending on the characteristics of the related oil. It can be noticed that for the oils ***A*** and ***B*** (UTTOs) there is an initial, rapid drop in viscosity (caused by intense transmission shear stress) which then remains quite stable, while the viscosity of ***C*** (hydraulic bio-based synthetic ester) keeps constant in a far longer test. ***D1*** and ***E1*** were refined vegetable oils added with antioxidant [4]. They underwent a progressive oxidation process from 40 to 50 h of work until the end of the test, which caused a progressive increase in viscosity, both at 40 °C and at high temperature. According to the diagram’s trends, such variations were contemporaneously detected by Lab data and sensors, which provided very similar trends of the curves of Lab-data and of ***S1_1_*** and ***S1_2_***, in both thermal conditions. Considering that in real working conditions the frequency of oil sampling for analysis is rather lower than in our test and that the laboratory needs time to provide its results, the sensor ***S1*** proved to be effective in detecting the variations in viscosity.

Increasing the concentration of antioxidant to the same vegetable oil bases provided the oils ***D2*** and ***E2*** whose performance was significantly improved, as their viscosity remained substantially stable up to about 275 h, when the increase started, although following a less marked trend than in ***D1*** and ***E1***. Moreover, in this case, the curves provided by laboratory and sensors had very similar, parallel trends. As mentioned above, the parallelism of the curves of Lab-data (taken as a reference) and of the sensors depends on the differences between the values obtained, point by point, in the two modes: since the chemical–physical characteristics of the oils vary type-by-type, they can differently affect the measurements carried out of by the sensors according to the factory pre-set calibration. Apart from this, what seems to be more important is the capability of ***S1*** sensors to monitor the viscosity variations, considering that one of the basic criteria to assess oil oxidation stability indicates a 20% increase in the viscosity at 40 °C as the limit for oil replacement (ISO 4263-3: 2015) [36]. In Table 11, the variations in viscosity observed at the end of each oil test are reported, both absolute and in percentage. The ***S1*** percent variations are sometimes higher and sometimes lower than those of the laboratory, but they are always of the same order of magnitude. The presence of such sensors in a plant would be therefore useful to continuous monitoring, to promptly detect any variations in viscosity measurement. The use of sample analysis would be greatly reduced and limited to punctual analyses to dispel any doubts about the correctness of the variations detected by the sensors.

The series of measurements provided by ***S1*** and by the laboratory underwent the test of Pearson. The resulting coefficients of correlation “***r***” and the probability “*p*” of uncorrelation are also reported in Table 11.

The results of the test of Pearson confirmed the above considerations, with high correlation between lab and sensor series of values during of all oils tests except for “***C***”. In this case, the low or negative correlation of data is explained by the very small viscosity variation, which testify that the viscosity remined substantially stable during more than 400 h in both lab and sensor series, as can be observed in Figure 8 (oil “***C***”).

### 3.3. Permittivity

The permittivity was measured by the sensors ***S1_1_*** (T = 40 °C), ***S1_2_*** (T = 88 °C), ***S2*** (T = 60 °C), and ***S4*** (T = 55 °C). The trends of the permittivity provided by the sensors were compared to the lab analyses results of ***PV*** and ***TAN*** to assess the capability of permittivity to detect the presence of any primary or secondary oxidation processes. The average extended uncertainty in permittivity measurements (Table 10) resulted lower than in lab determination of ***TAN*** and ***PV*** (Table 9). The trends of the measurements are shown in Figure 9. The diagrams of ***PV*** and ***TAN*** are reported in the left column, while those of the permittivity are in the central column. The missing Lab-data indicate the tests in which the relative analyses were not carried out, such as for ***PV*** in oils ***A*** and ***B***. The lack of the data of a particular sensor means that it had not yet been installed or that some problem occurred in the acquisitions system.

The diagrams in Figure 9 show that the ***TAN*** never had relevant variations but, in the oil, ***D1***, from an initial value of about 1.8 mg KOH g^−1^, it started to increase after 40 h and rose up to 2.7 mg KOH g^−1^. “***D1***” was one of the vegetable oils with lower concentration of antioxidant, and the same diagram shows that the increase in ***TAN*** was preceded by an increase in ***PV*** (started at about 20 h), which means that both primary and secondary oxidation processes were occurring in ***D1***.

Despite the differences among the values the sensors provided, their permittivity curves are substantially parallel, and their trends start to increase contemporaneously to the PV/TAN trends, similar to what was observed for the viscosity (see Section 3.2). With reference to the beginning of the test, the permittivity measured at the end of test increased by 11.56% and 11.58%, respectively for ***S1_1_*** (40 °C) and ***S2*** (60 °C), while for ***S1_2_*** (88 °C) the variation resulted in being slightly lower (10.28%). On the contrary, for the permittivity calculated at 40 °C by ***S2***, the variation was slightly higher (12.1%). Therefore, there is concordance between the laboratory results and sensors. According to the recommendations reported in Section 2.1 about the permittivity, a hypothetic detecting the above variations in a working plant should induce the checking of the oil’s condition, and probably to replace it.

The considerations made for the permittivity in ***D1*** can be extended to the tests with “***E1***” and “***E2***”, i.e., the tests with the same vegetable oil that respectively lasted 150 h and 360 h due to the presence in “***E2***” of a higher antioxidant concentration. In Figure 9, relevant permittivity variations can be observed in these tests. In these cases, the increase in permittivity accompanied the increase in ***PV***, as the ***TAN***, apart from some oscillations, remained stable in both cases. In ***E1*** the ***PV*** variation started around 40 h of work, as the permittivity started to increase at about 50 h, when the peroxide value became relevant (≅100 mEq O_2_ kg^−1^). In ***E1***, the curves of permittivity of ***S1_1_*** (40 °C), ***S2*** (60 °C), and ***S1_2_*** (88 °C) were parallel, and the variations referred to the test start value were respectively 0.32 mg KOH g^−1^ (10.25%), 0.31 mg KOH g^−1^ (10.16%), and 0.26 mg KOH g^−1^ (8.78%). The curve of the permittivity calculated at 40 °C by ***S2*** appears very irregular probably due to some problem that occurred in the sensor. At 100 h of test, the sensor ***S3*** was installed and had begun to measure the permittivity (at 55 °C), showing an increasing trend someway similar to the previous ones. In ***E2***, the sensors detected the oil alteration at about 250 h of work, corresponding to the sampling time of the oil that showed an increase in ***PV*** in lab analyses. In this case, all sensors had similar trends, with constant differences among the values provided point by point, which determined the parallelism of their curves that substantially follow the trend of the ***PV*** in the relative diagram. The percent variations in permittivity were of about 5% for all sensors.

As previously said, the ***TAN*** variations were also very small in the longer tests. For instance, in “***C***” the ***TAN*** started from 0.33 mg KOH g^−1^ and following a constant trend, it reached 0.62 mg KOH g^−1^ after 423 h, with an absolute variation of 0.29 mg KOH g^−1^, while, for ***TAN*** variations, the attention level was 2 mg KOH g^−1^. In this test, the permittivity measured by ***S1_1_*** (40 °C) and ***S2*** (60 °C) had similar behaviour. The increase was by 1.3% (0.04 in absolute value) in both cases, confirming the tendency of ***TAN***, despite the decreasing of ***PV*** whose values, however, were probably always too low to affect the permittivity.

The results of the test of Pearson to assess the correlation between the series of permittivity values of each sensor and those of ***TAN*** and ***PV*** are reported in Table 12. In the test with “***C***”, we can see a high positive correlation between permittivity and ***TAN*** and high negative correlation with ***PV***, which confirms the above comments to the diagrams of the same test ***C*** about the low ***PV*** level. High “***r***” values and very low “*p*” values can be observed in the tests on the oils ***D1***, ***E1***, and ***E2***, confirming that the sensors are suitable for monitoring the oil status relating to the risk of occurrence of any oxidation processes.

The test of Pearson was also carried out on the series of data provided by the sensors and indicated high correlation among the sensors in the same cases in which they were highly correlated to ***TAN*** or ***PV*** (tests on ***C***, ***D1***, ***E1***, and ***E2***”). Table 13 shows these results, with always high “***r***” values, while “*p*” resulted higher (*p* > 0.05) for ***S3***, which reflects some irregularities observed in Figure 9.

Although any detected variation in permittivity requires laboratory analysis of the samples in order to establish the causes and exactly quantify the alteration in progress, the use of sensors can help to significantly reduce the number of analyses and at the same time allow continuous monitoring of plants and machines in operating conditions. Beyond the generally similar parallel trends provided by the three sensors, ***S1*** measurements appeared more regular and reliable, including at high temperature, while some irregularities occurred to ***S2*** (***S2*** 60 °C in Figure 9), and less data were available for ***S3***. ***S1*** also showed the lowest uncertainty (Table 9). Eventually, it is important to underline that the temperature influences the permittivity. If oil temperature during monitoring is not constant, it will not be possible to correctly evaluate any permittivity variations. From this point of view, the ***S2*** function which calculates the permittivity at 40 °C from the permittivity at oil actual temperature, would be very useful in oil monitoring. However, due to several anomalous trends observed in the curves for the ***S2*** 40 °C tests on ***B***, ***D2***, and ***E1***, (Figure 9), its reliability needs to be verified in further tests.

### 3.4. Electric Conductivity

The electric conductivity (***E.C.***) was measured by ***S2*** at fluid operating temperature (60 °C). ***E.C.*** measurements were compared to those of ***TAN***, ***PV***, and ***RH***, parameters which could actually affect the electric conductivity of liquids. As regards the determination of ***Ū(x)_%_***, the manufacturer of ***S2***, declared that in the interval, 2000 pSi m^−1^ < E.C. < 800,000 pSi m^−1^, the sensor accuracy was 15 pSi m^−1^, i.e., ranging from 0.002% to 0.75%. For E.C. < 2000 pSi m^−1^, the accuracy decreased to 200 pSi m^−1^, which results in very high percent uncertainty. Therefore, in Table 10, ***Ū(x)_%_*** was reported only for E.C. > 2000 pSi m^−1^ (in the test with the oil ***D2***, ***E.C.*** always remained below 2000 pSi m^−1^). As for the permittivity, ***S2*** also provided the calculated values of ***E.C.*** referred to 40 °C. The series of ***E.C.*** values measured in the seven oil tests are shown in Figure 9 (right column diagrams). It can be noticed that in tests ***A*** and ***B***, the ***E.C.*** was much higher than in all other tests, probably due to specific characteristics and additives of the two UTTOs compared to those of hydraulic fluids and refined vegetable oils. On the contrary, ***Ū(x*)_%_** resulted lower in tests ***A*** and ***B***, with values respectively of 1.1% and 1.3%, compared to an average of 7.5% for the other oils. In general, similar to what was observed for the permittivity, the ***E.C.*** trends seem to follow those of ***TAN*** and ***PV*** and to well describe the start and progression of oil status alteration, also when ***E.C.*** *<* 2000 pSi m^−1^, with very high uncertainty level. The curves of ***E.C.*** calculated at 40 °C followed the expectably lower trends than those of ***E.C.*** measured at 60 °C only in oils ***A***, ***B***, ***C***, and ***E2***, while the anomalous trends observed in the other oils confirm the low reliability of calculated data.

The test of Pearson was carried out to verify the presence of correlation between ***E.C.*** (both measured, at 60 °C, and calculated, at 40 °C) and ***TAN*** and ***PV***. Moreover, since ***S2*** also measures the oil relative humidity (***RH***), the test of Pearson also regarded the possible correlation between ***EC*** and ***RH***. The results are reported in Table 14.

The results reported in Table 14 are similar to those of permittivity, with the highest “***r***” and lowest “*p*” values in correspondence of the most relevant variations of ***TAN*** and/or ***PV***, i.e., in the tests with oils ***C***, ***D1***, ***E1***, and ***E2***. As to the negative correlation between ***EC*** and ***PV***, it is similar to that observed for permittivity in the same test and can be explained in the same way. In the test with the oil “***B***”, the high “***r***” values were accompanied by “*p*” slightly higher than 0.05, which reduced the significance of the correlation. The ***RH*** values provided by ***S2*** are shown in the diagrams of Figure 11 (together with those of other sensors). The correlation between ***RH*** and ***EC*** is not evident in the seven tests carried out, probably because of the substantial stability of the humidity observed during all the work cycles against the variations of ***EC***, which was evidently caused by the increase in ***TAN*** and ***PV***. The relationship between oil ***RH*** values and the corresponding ***EC*** measurements will be deepened in subsequent specific tests.

### 3.5. Water Content

The presence of water in the oils was measured as parts per million (ppm) in laboratory analysis and as relative humidity, ***RH*** (%), by the sensors ***S2***, ***S3***, and ***S4***, at the temperature of the oil as it passes through each sensor, whose values were respectively 60 °C, 55 °C, and 40 °C. The differences depended on each sensor position and flowrate, differently set in order to achieve the most correct working conditions, which could favour the heat exchange between oil and external environment. As for the permittivity and the electric conductivity, ***S2*** also provided the ***RH*** calculated at a reference temperature that in this case was 20 °C. Said differences in oil temperatures affect the measurement of ***RH***. The diagram in Figure 10 shows the trends of ***RH*** recorded by the sensors during one day of test.

The oil heating initial phase does not appear in the diagram and the data refer to constant oil temperature in each sensor. Although the different ***RH*** levels provided by the sensors, their curves follow similar trends, slightly decreasing during the day as the oil progressively loses part of the water it contains (which increased before the daily test starting due to the night drop in temperature), until the equilibrium point. The ***RH*** calculated at 20 °C by ***S2*** is at the highest level (≅28%), while the ***RH*** at 60 °C, provided by the same sensor, is at the lowest (≅3%). The curve at 40 °C (by ***S4***) is immediately over (***RH*** ≅ 10%). The ***S3*** curve at 55 °C (***RH*** ≅ 26%) is anomalous because of its proximity to the ***S2*** curve at 20 °C. It can be noticed that the trend of the curve ***S4*** is less gradual than the others. From the observation of the recorded data, it resulted that the acquisition proceeds with a resolution of ±1.00 °C (resolution and accuracy were not reported in the data sheet o ***S4***). As the calculation of the uncertainty in measurement refers to constant temperature, ***RH*** resulted in being constant as well, with s = 0. As a consequence, ***Ū(x)_%_*** could not be provided for ***S4***.

The laboratory analyses were carried out on the samples of oils ***A***, ***B***, ***D1***, ***E1***, and ***E2*** using the Karl Fisher method (ISO 8534:2017). They were compared to the average daily ***RH*** values provided by the sensors recorded in the same days of oil sampling (Figure 11). The uncertainty in lab and sensors measurements are reported, respectively, in Table 9 and Table 10. In particular, Table 10 shows that, for ***S2***, ***Ū(x)_%_*** greatly varies from oil to oil and this is probably due to the generally low humidity levels in the oils, often below the 10% limit that defines the interval (10–90%) of highest ***S2*** accuracy. ***Ū(x)_%_*** appears more regular for ***S3***.

The comparison between absolute data as the water contents in ppm and relative data as ***RH*** is difficult because of the above cited influence of temperature on the ***RH*** level, the different units of measurement, and the risk of oil samples contamination by atmospheric water which could occur during the samplings or also in laboratory. However, the diagrams of Figure 11 provide some general indications:

− The trends of ***RH*** measured by the sensors ***S2***, ***S3,*** and ***S4*** reflect what was observed in Figure 10. Despite the different measured ***RH*** levels, they follow parallel trajectories indicating the same variations, peaks, etc. This common behaviour of the sensors contributes to accrediting the general correctness of the measurements provided and above all their usefulness in highlighting any anomalous trends of the measured parameters.
− The trend of ***RH*** calculated at 40 °C by ***S2*** is sometimes irregular (e.g., in ***C***, ***D1***, and ***E2*** oils) and differs from the other curves of ***RH*** confirming the above doubts about the reliability of the calculated values of permittivity and electric conductivity provided by ***S2***.
− The water contained in the oil is normally higher at the start of tests, and tends to decrease during the first period, then remaining substantially stable. Such a behaviour always occurred, except for ***B***, both in ***Water-KF*** and ***RH***.
− In the test with the oil ***A***, the ***Water-KF*** trend shows an anomalous peak, which is in contrast with the trends of the curves of the sensors. Such a peak is probably an outlier and in the subsequent statistical elaboration the series was analysed both with and without its corresponding value.
− Most differences between the curves of ***Water-KF*** and those of ***RH*** are sometimes caused by the different number of available data: e.g., in ***B***, ***D1***, ***E1***, and ***E2***, the number of analyses is far lower than the daily RH values. In the case of ***E2***, in which many more analyses have been carried out, a greater adherence of behaviour can be observed between the trends of water ***Water-KF*** and ***RH***.

Furthermore, in this case, the statistical analysis was based on the test of Person and aimed at verifying any correlation at first between the data from lab analyses and sensors measurements, then, among the sensors. The results of this comparison are reported in Table 15. In ***C*** and the lab analyses were not carried out, as in ***D2*** the measurements by ***S3*** started at about 80 h of test. In ***A*** and ***B*** no correlation appeared. In Table 15 appears the test ***A_1_***: here, the correlation analysis was made on the same dataset of the test ***A*** from which the ***Water-KF*** value at 60 h was eliminated as a probable outlier. The coefficients of correlation between sensors and Lab-data increased relating to “***A***”, but the probability of uncorrelation remained high. Among the other tests, we find very high correlation coefficients in ***D1*** and ***E1*** for all sensors data (except for the ***S2*_(calc@20 °C)_**) where, however, the probability of uncorrelation is higher than 0.05, probably due to the low number of data available for the test. Eventually, in ***E2***, in presence of more data than in previous tests, the correlation between ***Water-KF*** data and those provided by ***S2*_(60 °C)_**, ***S3*_(55 °C)_**, and ***S4*_(40 °C)_**, was very strong, with high “***r***” values corroborated by “*p*” always lower than 0.05. Moreover, in this case, the ***RH*** calculated at 20 °C was uncorrelated to the Lab-data.

The correlations between the data of sensors, considered pair by pair, are reported in Table 16. Strong and very strong correlations are more frequent now, often confirmed by very low “*p*” values, which reflect the parallel trends of the different ***RH*** curves observed in Figure 11. The cases of uncorrelation mostly occur in when the test regards the ***RH*** calculated at 20 °C by ***S2***.

The above results indicate that the sensors seem capable of following the trend of the humidity in the oils and could be conveniently installed in plants where the risk exists of oil contamination by water, such as in presence of oil–water heat exchangers, taking care that monitoring is carried out at oil constant temperature. The variability among the levels of ***RH*** detected by the sensors was probably caused by the different characteristics of both sensors and oils and by their interactions. Despite this, all sensors answer to ***RH*** variations in the same way. Based on the relating uncertainty in measurements, ***S3*** measurements appeared more reliable, while ***S4*** was less precise and accurate. The calculation of RH at 20 °C provided by ***S2*** was often irregular and does not seem reliable. As for the relationship between ***RH*** and the absolute water content, it could be clarified through specific tests that provide an adequate number of samples (and relative analysis results) and ***RH***, taking care to avoid any contamination of the samples by environmental water.

### 3.6. Particle Contamination

The level of particle contamination was determined in laboratory only for oils ***A*** an ***B*** at intervals of 30 h, while ***S4*** measurements were continuously carried out in all tests. Based on the number of particles detected in different dimensional classes, both methods provided the class of oil contamination according to the standards NAS 1638-2001 and ISO 4406:2021. Since no accuracy values were reported for the lab method and sensors, their uncertainty could not be calculated. Figure 12 shows the results for the seven oils according to the ISO standard: each test diagram reports the trends of the codes assigned to the oil in each of the three-dimensional classes “<4 μm”, “<6 μm”, and “<14 μm”. It can be noticed that, in the diagrams, the ISO code values provided by ***S4*** do not correspond to integers, but decimal numbers. This depends on the fact that, as said in “Section 2.7—Data collection and analysis”, all sensors’ data used in the comparison with Lab-data represented the averages of all daily measurements. The comparison between Lab-data and ***S4***-data was possible only for ***A*** and ***B***. In the relating diagrams we can observe that in all dimensional classes the curves of Lab-data are higher than those of ***S4***: in the class “<4 μm”, the curves of laboratory and of ***S4*** almost overlay; in the classes “<6 μm” and “<14 μm”, the distance progressively increases, which means a lower sensibility of ***S4*** towards bigger particles. However, all curves of the tests with oils ***A*** and ***B*** start from higher contamination values and follow similar decreasing trends, testifying the effectiveness of the filtration system of the test rig. Such decreasing trends are similar to the trends of the electric conductivity in the same oils (Figure 9). This suggests the possibility of a link between the particulate and the electric conductivity, given the absence of variations in other parameters potentially affecting the latter (e.g., TAN, PV, or RH). Such a link could be verified by investigating the nature of the particulate.

Despite said differences among the levels of the measurements, the test of Pearson applied to the paired data of laboratory and ***S2*** (Table 17) confirmed the strong (***r*** > 0.6) or very strong (***r*** > 0.8) correlation between their trends. The values of “*p*” resulted always lower than 0.05 except for the oil ***A***, in the class “>6 μm” (*p* = 0.103) and the oil ***B*** in the class “>14 μm” (*p* = 1.126).

Figure 12 also reports the diagrams of the levels of contamination provided by ***S4*** in the tests with ***C***, ***D1***, ***D2***, ***E1***, and ***E2*** where, in general, they remained stable and lower than in ***A*** and ***B***, except for ***E1***, which started from higher contamination levels in the three-dimensional classes and decreased during the test. Considering the substantially strong correlation between Lab-data and ***S4***-data, despite some differences in the contamination levels provided by the sensor, the latter seems capable to detect their variations and represents a useful device for in-line oil monitoring.

### 3.7. Ferromagnetic Particles

The results of lab determination of the iron (Fe) in the samples of the seven oils are reported in Table 18, together with the relating sampling times. In all samples the Fe amounts were below the detection threshold of the standard method adopted.

**Figure 13:**
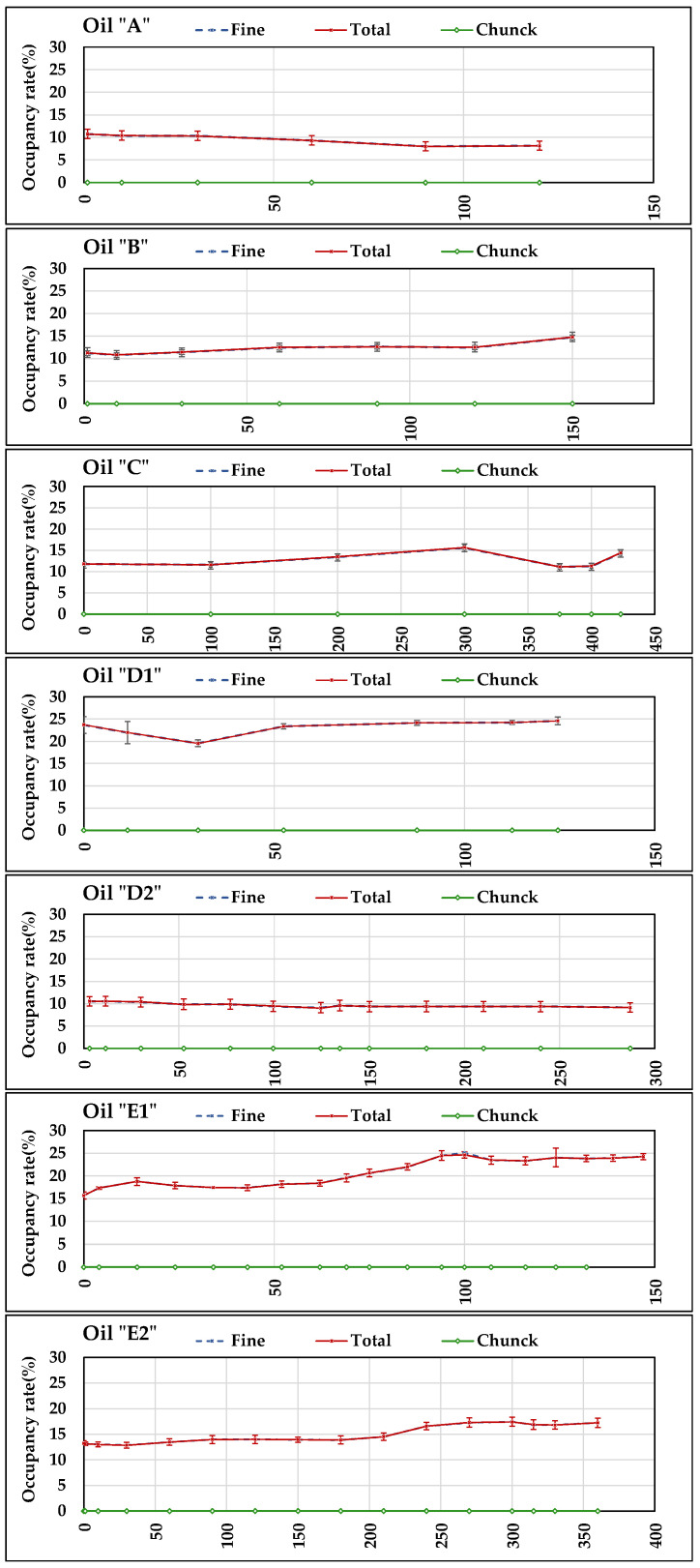
Presence of ferromagnetic particles detected by ***S5*** during the tests with the seven oils, described by the daily mean values of occupancy rate and relative standard deviations.

Figure 13 reports the diagrams of the daily averages of occupancy rate (fine fraction, chunk, and total) measured by ***S5*** in all days of tests. The accuracy of ***S5*** was not reported in the datasheet. However, the standard deviations of the daily measurements are reported in the diagrams. It can be noticed that all measurements relating to “fine fraction” and “total” start (at time 0 h) from values >0, which derive from the Fe particles retained by ***S5*** in previous tests, as explained in Section 2.2. Therefore, the occupancy rate in each test, referred to the starting value, in general seems to be rather stable with small variations positive or negative, which mainly occurred however in the oils that were less stable relating to the other measured parameters discussed above (***A***, ***B***, ***D1***, and ***E1***). The standard deviations values did not exceed 10% except in ***D1*** (second value) testifying a good stability in daily measurements. No coarse particles (chunk) were ever retained by ***S5*** magnetic head.

**Figure 14:**
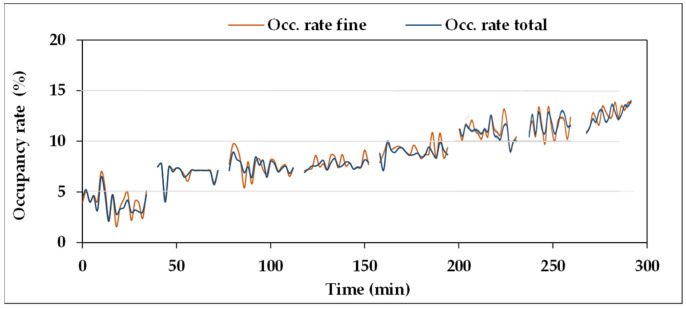
Variation of the occupancy rate consequent to increasing Fe_2_O_3_ concentration in the oil observed during the specific test with ***S5***.

In order to clarify the meaning of such behaviour, ***S5*** underwent a specific test, in the equipment of Figure 7, based on controlled conditions of Fe_2_O_3_ contamination of a mineral UTTO oil (density: 0.89 g mL^−1^). The trends of the occupancy rates at seven increasing levels of Fe_2_O_3_ contamination are reported in Figure 14 for “fine fraction” and “total”. Furthermore, in this case, the test started from an initial occupancy rate value >0 (about 5%), derived from previous tests, and progressively increased with further Fe additions to the oil. No chunks were detected.

The seven amounts of Fe_2_O_3_ powder added to the oil are reported in Table 19, together with the total amount obtained at each step, the resulting Fe concentrations (ppm), and the averages of the occupancy rates values of the eight measurements reported in Figure 14, both for “fine fraction” and “total”, whose values are very similar due to the absence of coarse particles to be summed to the fine fraction.

The diagram of Figure 14 and the data reported in Table 19 indicate that the occupancy rate increases with Fe_2_O_3_ concentration in the oil. The results of the test of Pearson confirm the presence of very strong correlation between the two variables, with very low “*p*” values. Therefore, ***S5*** could effectively concur with the monitoring of the status of plants and machines, through the detection of ferromagnetic particles, preventing any damage caused by wear of some components.

## 4. Conclusions

The subjects of this study were five sensors, from different manufacturers, used for in-line monitoring of the conditions of hydraulic fluids and lubricants. Through the measurements of proper physical parameters, the sensors should allow the detection of any variations in oil characteristics of lubricity during operations, helping to prevent plant damage. The five sensors were installed in a test rig designed for the testing of transmission lubricants and hydraulic fluids in severe work cycles under controlled conditions. The sensors were used to continuously monitor the conditions of seven oils of different origin during the related work cycles. The data they provided were compared to the results of laboratory analyses of oil samples periodically taken in order to verify the correlation between each physical parameter measured by the sensors and the oil characteristic they should monitor. The seven tests had different durations depending on the characteristics and behaviour of each oil. The test results provided the following indications:

− The test rig for lubricants and hydraulic fluids, which allow the application of controllable and repeatable work cycles, could be conveniently adopted as a basis for a methodology for sensors testing, aimed at assessing their accuracy and reliability as well as their behaviour with different oils.
− The sensors, for correct working, need particular attention to oil flowrate and line conformation in order to avoid that any turbulence interferes with the measure. For this reason, the sensors were installed in sections specially realized in the test rig, equipped with valves for the setting of the fluid flowrate, by-passable for sensors maintenance.
− Several parameters measured by the sensors are temperature dependant: the values of viscosity, permittivity, electric conductivity, and relative humidity vary with oil thermal conditions. As a consequence, in order to associate the trend of said parameters to any variations in oil characteristics, their observation must only concern the values referred to the same temperature conditions. In practical applications, after an initial heating phase, the oil reaches a thermal equilibrium resulting from the heating in the working section (e.g., high pressure circuits, and transmissions), the cooling, e.g., in heat exchangers and the room temperature. In such conditions of substantially stable temperature, the sensors data can be conveniently used. The presence, as in ***S2,*** of functions for calculating values at rated temperature from values at actual temperature would be very useful, simplifying the detection of trend variations. The test results showed that said functions behaved irregularly in several cases and need to be improved.

According to the previous general indications, the test results showed that, beyond some differences in accuracy and uncertainty in measurement, the variations in the physical parameters measured by the sensors were correlated to the variations in specific fluid characteristics measured in laboratory:

1. The dynamic viscosity provided by ***S1*** to the Kinematic Viscosity in the Hubbelode viscometer.
2. The permittivity (***S1***, ***S2***, ***S3***, and ***S4***) and the electric conductivity (***S2***) to variations of the TAN and/or PV.
3. The relative humidity (***S2***, ***S3***, and ***S4***) to the water content (ppm, KF method) when sufficient oil samples were available, as in oil “***E2***”.
4. The levels of particle contamination provided by ***S4*** to those determined in the laboratory.
5. The occupancy rate (***S5***) to the Fe_2_O_3_ concentration.

The correlation was strong or very strong in the presence of actual variations of oil characteristics due to the said alteration processes, while this was not evident in the oils that remained unaltered all along the test, which does not mean that the sensors did not work.

When variations occurred, they were promptly detected by the sensors and the trends of their data were confirmed by lab results. These, in normal operating conditions, are commonly provided some days after sampling. We must also consider that our comparison between sensors and lab data only refers to the days of oil samplings, where the sensors kept on measuring all the time. Moreover, in normal operating conditions, the frequency of sampling and lab analysis is far lower than in our study. In light of this, the sensors timeliness was very high relating to lab results and could significantly improve the quality of both oil conditions monitoring and prevention of plant damage. Strong correlations, which represent a sort of mutual validation, were also observed among the measurements of the same parameters provided by different sensors, i.e., permittivity (***S1***, ***S2***, ***S3***, and ***S4***) and relative humidity (***S2***, ***S3***, and ***S4***). Despite some differences in the levels of the measurements provided by different sensors, they follow parallel trends, detecting the same variations, when they occur.

The timeliness in detecting changes in the parameters they measure is a fundamental requirement for the sensors to be considered suitable for installation in plants and machines whose operation is based on the use of lubricants or hydraulic fluids, with the purpose of preventing damage caused by the loss of oil lubricating properties. Then, the choice of an appropriate set of sensors could cover a wide spectrum of parameters, granting a high protection of the system, which could be further expanded when combined with other in-line techniques (vibration analysis and thermal imaging).

Apart from fixed installations, where sensors are often already used, they could be usefully applied to mobile machines as well. For instance, in the agricultural sector, where tractors and implements carry out intense processing and their lubricants are exposed to uneasy environmental conditions (high temperatures and humidity, dust contamination, etc.), correctly installed sensors could contribute to safeguard the efficiency of the machines through the indications on the oil status. The installation and testing of a series of sensors on an agricultural tractor used in normal farming will be the subject of a further study.

## Figures and Tables

**Table 6:** Test conditions in the test rig. Values of the working parameters adopted in the work cycle.

Test Rig’s Section	Main Parameters of Working Conditions	Measure Unit	Work Cycle
Main circuit (low-pressure)	Oil volume	dm^3^	20
	Pressure level	MPa	<0.15
	Oil temperature ^1^	°C	<60
Hydraulic	Oil temperature ^2^	°C	≈100
	Flowrate	dm^3^ min^−1^	5.7
	Pressure level	MPa	40
	High-pressure pump speed	min^−1^	1800
	Maximum hydraulic power	kW	3.8
Transmission (speed multiplier)	Oil volume in the multiplier	dm^3^	2.6
	Oil temperature ^3^	°C	60
	Oil temperature ^4^	°C	≈87
	Flowrate ^5^	dm^3^ min^−1^	0.2
	Electric engine speed	min^−1^	660
	Dynamometric brake shaft speed	min^−1^	1670
	Torque at dynamometric brake ^6^	daNm	10.4
	Mechanical power ^6^	kW	18.2

^1^ In the reservoir/before high-pressure pump. ^2^ After the lamination at 40 MPa. ^3^ At transmission inlet. ^4^ At transmission outlet. ^5^ In the multiplier gearbox. ^6^ Depending on the electric engine characteristics.

**Table 7:** Main characteristics of the oils used in the tests.

Properties	UTTO		Hydraulic Oils				
	*A*	*B*	*C*	*D1*	*D2*	*E1*	*E2*
Origin	^1^	^2^	^2^	^3^	^3^	^3^	^3^
Physical state at 20 °C	Liquid	Liquid	Liquid and viscous				
Appearance and colour	Yellow-clear						
Pour point (°C)	−40	−33	−37	8	8	4	4
Viscosity at 40 °C (cSt)	60.4	56.0	42.8	49.3	49.3	34.5	34.5
Viscosity at 100 °C (cSt)	9.5	11.9	9.3	10.4	10.4	7.8	7.8
Viscosity index (N)	139	215	209	207	207	214	214
TAN (mg KOH g^−1^)	-	-	-	2.43	2.43	1.21	1.21
Flash point (°C)	210	>260	200	-	-	-	-
Density at 15 °C (g cm^−3^)	0.875	n.a.	0.89	0.91	0.91	0.9	0.9

^1^ Petrol based synthetic esters. ^2^ Bio-based synthetic esters. ^3^ Vegetable oil added with antioxidant.

**Table 8:** Sensors and reference Lab-data used with each oil.

		UTTO		Hydraulic Oils				
		*A*	*B*	*C*	*D1*	*D2*	*E1*	*E2*
Sensors	S1	-	-	x	x	x	x	x
	S2	x	x	x	x	x	x	x
	S3	x	x	x	x	x	x	x
	S4	-	-	x	x	x	x	x
	S5	x	x	x	x	x	x	x
Lab-data	CREA	-	-	x	x	x	x	x
	LMP srl	x	x	-	-	-	-	-
	MECOIL srl	x	x	-	-	-	-	-
	INNOVHUB	-	-	-	x	x	x	x

**Table 9:** Uncertainty in measurement declared by the laboratories which analysed the samples of the tested oils.

Parameter	Unit	Lab Measurements Accuracy (*p* = 95%)			
		Mecoil	LMP	INNOVHUB	CREA-IT
Kin. Viscosity@40 °C	% of value	±1	±1	-	±0.88
Kin. Viscosity@100 °C	% of value	±1	±1	-	±0.88
TAN	% of value	±5	±5	-	±1.5
PV	% of value	-	-	-	±1.2
Water (K.F method)	% of value	±5	-	±5	-

**Table 10:** Mean standard errors (SE) in the measurements of viscosity, permittivity, electric conductivity, and relative humidity (***RH***) carried out by the sensors ***S1***, ***S2***, ***S3***, and ***S4***.

Extended Uncertainty, *Ū(x)_%_* (±% of Value)									
Parameter	K.V._40 °C_ (cSt)	K.V._88 °C_ (cSt)	Permittivity			El. Cond. (pSi m^−1^)	RH (%)		
Sensor	*S1_1_*	*S1_2_*	*S1*	*S2* ^1^	*S3*	*S2* ^2^	*S2* ^3^	*S3*	*S4* ^4^
Oil *A*	9.54	8.98	1.33	3.10	-	1.08	16.12	10.46	11.06
Oil *B*	6.18	-	1.14	2.77	-	1.32	11.24	7.36	6.22
Oil *C*	7.58	7.32	1.25	2.44	7.56	6.48	52.62	12.44	6.66
Oil *D1*	9.44	6.96	1.40	2.00	6.38	6.60	37.86	3.38	0.00
Oil *D2*	7.06	5.62	1.92	2.08	5.78	7.78	25.32	7.68	0.02
Oil *E1*	8.10	6.08	1.22	2.01	5.80	8.30	23.32	13.50	0.74
Oil *E2*	8.40	5.80	2.54	2.12	5.66	9.10	13.92	9.74	0.00

^1^ Since in our case, the permittivity is always ≅3, an accuracy of ±0.015 (Table 1) means ≅0.5%. ^2^ Only the Electric Conductivity in the interval 2000–800,000 pSI m^−1^ was considered, where the accuracy was ±10 pSi m^−1^, used to calculate the % accuracy from average daily measurements (Table 2). The El. Cond. <2000 pSi m^−1^ was not considered because of the too low accuracy (±200 pSi m^−1^). ^3^ The accuracy in the interval 10–90% is ±3%. Outside said interval the accuracy is ±5%. ^4^ Accuracy not declared for RH.

**Table 11:** Absolute and percent variations in Kinematic Viscosity at the end of each oil test and correlation coefficients “***r***” between laboratory and ***S1*** measurements (*p*: probability of uncorrelation). The bold, underlined values respectively refer to *r* > 0.60 and *p* < 0.05.

Oils		Temperature of Measurement	Δ Viscosity (cSt)		Δ Viscosity (%)		Correlation Lab/*S1*	
			Lab.	*S1*	Lab.	*S1*	*r*	*p*
UTTO	*A*	40 °C	7.00	4.32	−13.21	−10.10	**0.94**	***4.7 × 10^−3^***
		100 °C	1.10	0.93	−12.94	−9.82	**0.96**	***2.5 × 10^−3^***
	*B*	40 °C	3.53	2.48	−8.68	−6.50	**0.98**	***4.3 × 10^−4^***
		100 °C	-	-	-	-	-	-
Hydraulic	*C*	40 °C	1.32	2.65	−1.64	4.05	0.48	*2.3 × 10^−1^*
		100 °C	0.38	0.55	−3.86	4.98	−0.47	*2.4 × 10^−1^*
	*D1*	40 °C	30.00	42.68	62.67	95.41	**1.00**	***6.4 × 10^−13^***
		100 °C	3.86	4.09	37.19	35.40	**0.99**	***7.0 × 10^−5^***
	*D2*	40 °C	4.87	2.59	13.55	5.49	**0.89**	***1.4 × 10^−3^***
		100 °C	0.67	1.00	7.74	9.22	**0.92**	***4.6 × 10^−4^***
	*E1*	40 °C	26.17	19.82	75.39	62.00	**0.99**	***1.2 × 10^−6^***
		100 °C	4.39	4.23	53.70	44.60	**0.95**	***3.2 × 10^−4^***
	*E2*	40 °C	12.23	12.91	33.04	38.89	**0.96**	***3.1 × 10^−9^***
		100 °C	1.58	1.72	18.12	19.49	**0.88**	***6.9 × 10^−6^***

**Table 12:** Correlation coefficients “***r***” and probability of uncorrelation *“p”* (in Italic) between permittivity measured by the sensors and the ***TAN*** and ***PV*** values resulting from laboratory analysis. The bold, underlined values refer to *r* > 0.60. The bold, double-underlined values refer to *r* < −0.6. The bold, underlined values refer to *p* < 0.05.

Oils	Lab. Param.	*S1_1_* (40 °C)		*S1_2_* (88 °C)		*S2* (60 °C)		*S2* (40 °C)		*S3* (55 °C)	
		*r*	*p*	*r*	*p*	*r*	*p*	*r*	*p*	*r*	*p*
*A*	*TAN*	−0.14	*7.9 × 10^−1^*	−0.22	*6.7 × 10^−1^*	−0.31	*5.4 × 10^−1^*	0.36	*4.8 × 10^−1^*	-	-
	*PV*	-	-	-	-	-	-	-	-	-	-
*B*	*TAN*	0.31	*5.6 × 10^−1^*	-	-	0.00	*1.0*	**−0.83**	* **4.2 × 10^−2^** *	-	-
	*PV*	-	-	-	-	-	-	-	-	-	-
*C*	*TAN*	**0.81**	***1.4 × 10^−2^***	-	-	**0.98**	***1.9 × 10^−6^***	-	-	-	-
	*PV*	**−0.84**	***8.5 × 10^−3^***	-	-	**−0.98**	***2.1 × 10^−5^***	-	-	-	-
*D1*	*TAN*	**0.97**	***2.0 × 10^−8^***	**0.97**	***2.7 × 10^−8^***	**0.95**	***1.1 × 10^−6^***	**0.92**	***6.5 × 10^−6^***	-	-
	*PV*	**0.91**	***1.5 × 10^−5^***	**0.91**	***2.0 × 10^−5^***	**0.91**	***2.1 × 10^−5^***	**0.92**	***8.0 × 10^−6^***	-	-
*D2*	*TAN*	−0.14	*6.5 × 10^−1^*	−0.47	*1.1 × 10^−1^*	0.00	*1.0*	−0.43	*1.5 × 10^−1^*	−0.46	*1.5 × 10^−1^*
	*PV*	0.17	*5.7 × 10^−1^*	−0.29	*3.4 × 10^−1^*	0.00	*1.0*	−0.58	***3.6 × 10^−2^***	−0.53	*6.2 × 10^−2^*
*E1*	*TAN*	0.07	*7.8 × 10^−1^*	0.19	*4.7 × 10^−1^*	−0.06	*8.2 × 10^−1^*	−0.07	*7.9 × 10^−1^*	**0.94**	***2.0 × 10^−3^***
	*PV*	**0.94**	***3.6 × 10^−9^***	**0.92**	***1.1 × 10^−7^***	0.38	*1.2 × 10^−1^*	**0.62**	***5.8 × 10^−3^***	**0.93**	***2.6 × 10^−3^***
*E2*	*TAN*	−0.12	*6.7 × 10^−1^*	−0.20	*4.7 × 10^−1^*	−0.18	*5.3 × 10^−1^*	−0.21	*4.6 × 10^−1^*	−0.19	*5.0 × 10^−1^*
	*PV*	**0.96**	***7.4 × 10^−9^***	**0.98**	***1.9 × 10^−10^***	**0.97**	***2.5 × 10^−9^***	**0.96**	***2.0 × 10^−8^***	0.82	***1.7 × 10^−4^***

**Table 13:** Correlation coefficients “***r***” (in Italic) and probability “*p*” of uncorrelation between the series of permittivity values provided by the sensors in the tests with oils ***C***, ***D1***, ***E1***, and ***E2***. The bold underlined values respectively refer to *r* > 0.60 and *p* < 0.05.

*r*\*p*		*S1_1_* (40 °C)	*S1_2_* (88 °C)	*S2* (60 °C)	*S2* (40 °C)	*S3* (55 °C)
*C*	*S1_1_* (40 °C)			**1.48 × 10^−2^**		
	*S1_2_* (88 °C)					
	*S2* (60 °C)	***0.81***				
	*S2* (40 °C)					
	*S3* (55 °C)					
*D1*	*S1_1_* (40 °C)		**3.92 × 10^−11^**	**2.93 × 10^−5^**	**1.72 × 10^−4^**	
	*S1_2_* (88 °C)	***0.998***		**3.40 × 10^−5^**	**2.80 × 10^−4^**	
	*S2* (60 °C)	***0.98***	***0.98***		**2.64 × 10^−3^**	
	*S2* (40 °C)	***0.98***	***0.97***	***0.96***		
	*S3* (55 °C)					
*E1*	*S1_1_* (40 °C)		**1.2 × 10^−9^**	**8.9 × 10^−10^**	**3.4 × 10^−3^**	2.4 × 10^−1^
	*S1_2_* (88 °C)	***0.98***		**1.0 × 10^−8^**	**6.1 × 10^−3^**	5.2 × 10^−1^
	*S2* (60 °C)	***0.98***	***0.97***		**7.9 × 10^−3^**	**4.0 × 10^−3^**
	*S2* (40 °C)	***0.89***	***0.88***	***0.88***		**1.5 × 10^−3^**
	*S3* (55 °C)	***0.99***	***0.96***	***0.91***	***0.94***	
*E2*	*S1_1_* (40 °C)		**1.7 × 10^−8^**	**1.9 × 10^−7^**	**3.5 × 10^−6^**	6.1 × 10^−1^
	*S1_2_* (88 °C)	***0.99***		**7.0 × 10^−13^**	8.3 × 10^−2^	8.3 × 10^−2^
	*S2* (60 °C)	***0.99***	***0.997***		5.9 × 10^−1^	5.9 × 10^−1^
	*S2* (40 °C)	***0.98***	***0.99***	***0.99***		**1.6 × 10^−4^**
	*S3* (55 °C)	***0.85***	***0.84***	***0.85***	***0.82***	

**Table 14:** Correlation coefficients “***r***” and probability “*p*” of uncorrelation (in Italic) between **E.C.** and ***TAN, PV,*** and ***RH***. The bold, underlined values refer to *r* > 0.60. The bold, double-underlined values refer to *r* < −0.6. The bold, underlined values refer to *p* < 0.05.

Oil Test	*r*\*p*	E.C.–TAN		E.C.–PV		E.C.–RH	
		E.C._(60 °C)_	E.C._(40 °C)_	E.C._(60 °C)_	E.C._(40 °C)_	E.C._(60 °C)_	E.C._(40 °C)_
*A*	*r*	−0.22	0.1	-	-	0.04	−0.08
	*p*	*6.76 × 10^−1^*	*8.44 × 10^−1^*	-	-	*9.44 × 10^−1^*	*8.84 × 10^−1^*
*B*	*r*	**0.87**	**0.86**	-	-	**−0.69**	−0.59
	*p*	*5.72 × 10^−2^*	*5.90 × 10^−2^*	-	-	*1.95 × 10^−1^*	*2.98 × 10^−1^*
*C*	*r*	**0.94**	**0.92**	**−0.92**	**−0.89**	0.13	0.12
	*p*	***6.50 × 10^−4^***	***1.37 × 10^−3^***	***1.42 × 10^−3^***	***3.01 × 10^−3^***	*7.53 × 10^−1^*	*7.80 × 10^−1^*
*D1*	*r*	**0.92**	**0.72**	**0.98**	**0.93**	**0.63**	0.46
	*p*	***7.73 × 10^−6^***	***6.01 × 10^−3^***	***2.09 × 10^−9^***	***2.88 × 10^−6^***	***2.02 × 10^−2^***	*1.12 × 10^−1^*
*D2*	*r*	**0.76**	**0.7**	**0.75**	**0.72**	−0.46	−0.5
	*p*	***2.57 × 10^−3^***	***7.99 × 10^−3^***	***3.14 × 10^−3^***	***5.46 × 10^−3^***	*1.12 × 10^−1^*	*8.29 × 10^−2^*
*E1*	*r*	−0.08	−0.05	**0.94**	**−0.62**	0.16	0.1
	*p*	*7.75 × 10^−1^*	*8.44 × 10^−1^*	***1.19 × 10^−8^***	***4.68 × 10^−3^***	*5.28 × 10^−1^*	*6.92 × 10^−1^*
*E2*	*r*	−0.24	−0.14	**0.95**	**0.97**	−0.15	−0.08
	*p*	*3.85 × 10^−1^*	*6.15 × 10^−1^*	***4.47 × 10^−8^***	***3.67 × 10^−9^***	*5.83 × 10^−1^*	*7.68 × 10^−1^*

**Table 15:** Correlation coefficients “***r***” and probability “*p*” of uncorrelation (in Italic) between the trend of water content provided by lab analysis (Karl Fisher method) and the trends of relative humidity (***RH***) provided by ***S2***, ***S3***, and ***S4*** during the tests with the seven oils. The bold, underlined values respectively refer to *r* > 0.60 and *p* < 0.05.

Oils	*r*\*p*	Water-KF			
		*S2*_(60 °C)_	*S2*_(calc@20 °C)_	*S3*_(55 °C)_	*S4*_(40 °C)_
*A*	*r*	−0.18	−0.16	−0.38	−0.48
	*p*	*7.3 × 10^−1^*	*7.6 × 10^−1^*	*4.6 × 10^−1^*	*3.3 × 10^−1^*
*A1* ^1^	*r*	**0.62**	**0.74**	0.51	0.51
	*p*	*2.7 × 10^−1^*	*1.6 × 10^−1^*	*3.8 × 10^−1^*	*3.8 × 10^−1^*
*B*	*r*	−0.06	−0.30	0.40	−0.32
	*p*	*9.0 × 10^−1^*	*5.2 × 10^−1^*	*5.0 × 10^−1^*	*4.9 × 10^−1^*
*D1*	*r*	**0.99**	0.31	-	**0.86**
	*p*	*9.6 × 10^−2^*	*8.0 × 10^−1^*	-	*3.5 × 10^−1^*
*E1*	*r*	**0.88**	**0.62**	**0.90**	**0.98**
	*p*	*1.2 × 10^−1^*	*3.8 × 10^−1^*	*2.8 × 10^−1^*	***2.4 × 10^−2^***
*E2*	*r*	**0.90**	−0.13	**0.90**	**0.81**
	*p*	***1.3 × 10^−2^***	*8.1 × 10^−1^*	***1.3 × 10^−2^***	***4.9 × 10^−2^***

^1^ Correlation analysis carried out on the dataset of the oil ***A***: in ***A1*** the data at 60 h of test were not considered as the value 494 ppm is probably an outlier.

**Table 16:** Correlation coefficients “***r***” and probability “*p*” (in Italic) of uncorrelation among the trends of relative humidity (***RH***) provided by ***S2***, ***S3***, and ***S4*** during the tests with the seven oils. The bold, underlined values respectively refer to *r* > 0.60 and *p* < 0.05.

Oil Test	*r*\*p*	*S2*_(60 °C)_			*S2*_(calc@20 °C)_			*S3*_(55 °C)_			*S4*_(40 °C)_		
		*S2*_(calc@20 °C)_	*S3*_(55 °C)_	*S4*_(40 °C)_	*S2*_(60 °C)_	*S3*_(55 °C)_	*S4*_(40 °C)_	*S2*_(60 °C)_	*S2*_(calc@20 °C)_	*S4*_(40 °C)_	*S2*_(60 °C)_	*S2*_(calc@20 °C)_	*S3*_(55 °C)_
*A*	*r*	**0.85**	**0.85**	**0.93**	**0.85**	**0.96**	**0.88**	**0.85**	**0.96**	**0.94**	**0.93**	**0.88**	**0.94**
	*p*	***3.2 × 10^−2^***	***3.1 × 10^−2^***	***6.9 × 10^−3^***	***3.2 × 10^−2^***	***2.7 × 10^−3^***	***2.0 × 10^−2^***	***3.1 × 10^−2^***	***2.7 × 10^−3^***	***4.8 × 10^−3^***	***6.9 × 10^−3^***	***2.0 × 10^−2^***	***4.8 × 10^−3^***
*B*	*r*	0.51	**0.84**	**0.96**	0.51	0.53	0.56	**0.84**	0.53	**0.78**	**0.96**	0.56	0.78
	*p*	***2.9 × 10^−2^***	***2.1 × 10^−3^***	***1.7 × 10^−6^***	***2.9 × 10^−2^***	***1.2 × 10^−1^***	***1.7 × 10^−2^***	***2.1 × 10^−3^***	*1.2 × 10^−1^*	***7.7 × 10^−3^***	***1.7 × 10^−6^***	***1.7 × 10^−2^***	***7.7 × 10^−3^***
*C*	*r*	**0.63**	**0.89**	**0.92**	**0.69**	**0.81**	**0.85**	**0.89**	**0.78**	**0.98**	**0.92**	**0.82**	**0.98**
	*p*	*1.3 × 10^−1^*	***7.7 × 10^−3^***	***3.3 × 10^−3^***	*5.7 × 10^−1^*	***1.4 × 10^−2^***	***7.4 × 10^−3^***	***7.7 × 10^−3^***	***3.9 × 10^−2^***	***1.6 × 10^−4^***	***3.3 × 10^−3^***	***2.3 × 10^−2^***	***1.6 × 10^−4^***
*D1*	*r*	**0.62**	-	**0.94**	**0.62**	-	**0.80**	-	-	-	**0.94**	**0.80**	-
	*p*	***3.1 × 10^−2^***	-	***5.0 × 10^−2^***	***3.1 × 10^−2^***	-	***1.9 × 10^−3^***	-	-	-	***5.0 × 10^−2^***	***1.9 × 10^−3^***	-
*D2*	*r*	**0.73**	**0.81**	**0.85**	**0.73**	**0.65**	**0.76**	**0.81**	**0.65**	**0.91**	**0.85**	**0.76**	**0.91**
	*p*	***4.9 × 10^−3^***	***7.3 × 10^−4^***	***2.0 × 10^−4^***	***4.9 × 10^−3^***	***1.7 × 10^−2^***	***2.7 × 10^−3^***	***7.3 × 10^−4^***	***1.7 × 10^−2^***	*1.8 × 10^−1^*	***2.0 × 10^−4^***	***2.7 × 10^−3^***	*1.8 × 10^−1^*
*E1*	*r*	**0.71**	**0.81**	**0.89**	**0.67**	0.36	0.48	**0.81**	0.54	**0.90**	**0.89**	**0.81**	**0.90**
	*p*	*7.1 × 10^−2^*	***2.7 × 10^−2^***	***6.9 × 10^−3^***	***1.7 × 10^−3^***	*3.4 × 10^−1^*	***3.6 × 10^−2^***	***2.7 × 10^−2^***	*2.1 × 10^−1^*	***5.9 × 10^−3^***	***6.9 × 10^−3^***	***2.8 × 10^−2^***	***5.9 × 10^−3^***
*E2*	*r*	−0.01	**0.97**	**0.87**	−0.01	−0.01	0.14	**0.97**	−0.01	**0.86**	**0.87**	0.13	**0.86**
	*p*	*9.8 × 10^−1^*	***2.0 × 10^−6^***	*1.5 × 10^−1^*	*9.8 × 10^−1^*	*9.6 × 10^−1^*	*6.3 × 10^−1^*	***2.0 × 10^−6^***	*9.6 × 10^−1^*	***1.8 × 10^−2^***	*1.5 × 10^−1^*	*6.3 × 10^−1^*	***1.8 × 10^−2^***

**Table 17:** Correlation coefficients “***r***” and probability “*p*” of uncorrelation (in Italic) among the trends of the ISO 4406:2021 particle contamination codes showed in Figure 12. The bold, underlined values respectively refer to *r* > 0.60 and *p* < 0.05.

Oil	*r*\*p*	>4 μm	>6 μm	>14 μm
*A*	*r*	**0.96**	**0.73**	**0.81**
	*p*	***0.002***	*0.103*	***0.049***
*B*	*r*	**0.80**	**0.81**	**0.63**
	*p*	***0.031***	***0.026***	*0.126*

**Table 18:** Fe contents in oils samples at different working times, resulting from lab analyses.

Oil Test	Fe Content (ppm)/Working Time (h)							Lab
*A*	<1/0	<1/10	<1/30	<1/60	<1/90	-	<1/120	MECOIL
*B*	<1/0	<1/10	<1/30	<1/60	<1/90	<1/120	<1/150	MECOIL
*C*	-	-	-	-	-	-	-	-
*D1*	<1/0	-	-	<1/60	-	-	<1/140	InnovHub
*D2*	<1/0	<1/52	<1/134	-	<1/180	-	<1/287	InnovHub
*E1*	<1/0	-	<1/90	-	<1/100	-	<1/148	InnovHub
*E2*	<1/0	<1/60	<1/180	<1/270	<1/315	<1/375	<1/150	InnovHub

**Table 19:** Results of the specific Fe_2_O_3_ contamination test carried out on ***S5***.

Step	Fe_2_O_3_ Added to the Oil			Occupancy Rate	
	Massa Added	Total Mass	Fe Conc.	Fine Fraction	Total
	mg	mg	ppm	%	%
0	*0*	*0*	0.00	3.90	4.07
1	*1.3*	*1.3*	1.46	6.88	6.90
2	*1.7*	*3*	3.37	7.52	7.60
3	*2.2*	*5.2*	5.84	7.65	7.80
4	*3.6*	*8.8*	9.89	8.90	9.20
5	*4.5*	*13.3*	14.94	10.80	10.60
6	*4.5*	*17.8*	20.00	11.60	11.50
7	*5.5*	*23.3*	26.18	12.50	12.30
**Correlation Occ. rate/Fe concentration**			***r***	0.946	0.943
			***p***	0.0004	0.0004
